# Filopodia-based contact stimulation of cell migration drives tissue morphogenesis

**DOI:** 10.1038/s41467-020-20362-2

**Published:** 2021-02-04

**Authors:** Maik C. Bischoff, Sebastian Lieb, Renate Renkawitz-Pohl, Sven Bogdan

**Affiliations:** 1 Institute of Physiology and Pathophysiology, Department of Molecular Cell Physiology, Philipps-University, Marburg, Germany; 2 Computer Graphics and Multimedia Programming, Philipps-University, Marburg, Germany; 3 Developmental Biology, Philipps-University, Marburg, Germany

**Keywords:** Cell biology, Cell adhesion, Cell migration, Cell polarity, Cytoskeleton

## Abstract

Cells migrate collectively to form tissues and organs during morphogenesis. Contact inhibition of locomotion (CIL) drives collective migration by inhibiting lamellipodial protrusions at cell–cell contacts and promoting polarization at the leading edge. Here, we report a CIL-related collective cell behavior of myotubes that lack lamellipodial protrusions, but instead use filopodia to move as a cohesive cluster in a formin-dependent manner. We perform genetic, pharmacological and mechanical perturbation analyses to reveal the essential roles of Rac2, Cdc42 and Rho1 in myotube migration. These factors differentially control protrusion dynamics and cell–matrix adhesion formation. We also show that active Rho1 GTPase localizes at retracting free edge filopodia and that Rok-dependent actomyosin contractility does not mediate a contraction of protrusions at cell–cell contacts, but likely plays an important role in the constriction of supracellular actin cables. Based on these findings, we propose that contact-dependent asymmetry of cell–matrix adhesion drives directional movement, whereas contractile actin cables contribute to the integrity of the migrating cell cluster.

## Abstract (web summary)

Contact stimulation of migration drives tissue morphogenesis. Here the authors report that filopodia-based contact-dependent asymmetry of cell–matrix adhesion drives directional movement, whereas contractile actin cables contribute to the integrity of the migrating cell cluster in the myotubes of *Drosophila* developing testes.

## Introduction

The ability of cells to migrate as a collective is crucial during tissue morphogenesis and remodeling^1,2^. The molecular principles of collective cell migration share features with the directed migration of individual cells. The major driving forces in migrating single cells are Rac-mediated protrusions of lamellipodia at the leading edge, formed by Arp2/3 complex-dependent actin filament branching and Rho-dependent actomyosin-driven contraction at the cell rear^3,4^. Cells can migrate directionally in response to a variety of chemical cues, recognized by cell surface receptors that initiate downstream signaling cascades controlling the activity or recruitment of Rho GTPases. Directional cell locomotion is also controlled by mechanical stimuli such as upon cell–cell contact^5–7^. A well-known phenomenon is contact inhibition of locomotion (CIL), whereby two colliding cells change direction after coming into contact^8,9^. Roycroft and Mayor provided first mechanistic evidence how CIL might act in vivo as the driving force to polarize neural crest cells that derived from the margin of the neural tube and disperse by migration during embryogenesis^10,11^.

In neural crest cells, CIL involves distinct stages of cell behavior including cell–cell contact, protrusion inhibition, repolarization, contraction, and migration away from the collision^12^. The initial cell–cell contact requires the formation of transient cadherin-mediated cell junctions. Once the cells come in close contact, a disassembly of cell–matrix adhesion near the cell–cell contact and the generation of new cell–matrix adhesions at the free edge occur. Such mechanical crosstalk between N-cadherin-mediated cell–cell adhesions and integrin-dependent cell–matrix adhesions has been recently described in vivo during neural crest cell migration in both *Xenopus* and zebrafish embryos^13^. However, the loss of cell–matrix adhesions at cell contacts alone is not sufficient to drive CIL. A subsequent repolarization of the cells away from the cell–cell contact and thereby the generation of new cell–matrix adhesions and protrusions at the free edge are required to induce cell migration away from the collision. In neural crest cells, this depends on the polarized activity of the two Rho GTPases, Rac1 and RhoA^14^. A model of CIL has been proposed in which a contact-dependent intracellular Rac1/RhoA gradient is formed that generates an asymmetric force driving directed cell migration^15^. N-cadherin binding triggers a local increase of RhoA and inhibits Rac1 activity at the site of contact^14,16^. Thus, Rac1-dependent protrusions become biased to the opposite end of the cell–cell contact and cells migrating away from the collision.

Overall, CIL has been successfully used to explain contact-dependent collective migration of loose clusters of mesenchymal cells such as neural crest cells and hemocytes^12^, but it is still unclear whether mechanisms governing CIL might also contribute to the migratory behavior of cohesive cell clusters or epithelia^5,7^.

Here, using an integrated live-cell imaging and genetic approach, we identified a CIL-related, contact-dependent migratory behavior of highly cohesive nascent myotubes of the *Drosophila* testis. Myotubes lack lamellipodial cell protrusions, but instead form numerous large filopodia generated at both N-cadherin-enriched cellular junctions at cell–cell contacts and integrin-dependent cell–matrix sites at their free edge. Filopodia-based myotube migration requires formins and the Rho family small GTPases Rac2, Cdc42, and RhoA, whereas the Arp2/3 complex and its activator, the WAVE regulatory complex (WRC), seem only to contribute to filopodia branching. Rac2 and Cdc42 differentially control not only protrusion dynamics but also cell–matrix adhesion formation. Unlike CIL, RhoA is not activated at cell–cell contacts, but rather gets locally activated along retracting protrusions. Genetic and pharmacological perturbation analysis further revealed an important requirement of Rho/Rok-driven actomyosin contractility in myotube migration.

In summary, we propose a model in which N-cadherin-mediated contact-dependent asymmetry of cell–matrix adhesion acts as a major switch to drive cell movement toward the free space, whereas contractile actin cables contribute to the integrity of the migrating cell cluster.

## Results

### Long-term live imaging of *Drosophila* smooth-like testes muscles as a collective cell migration model

**Fig. 1:**
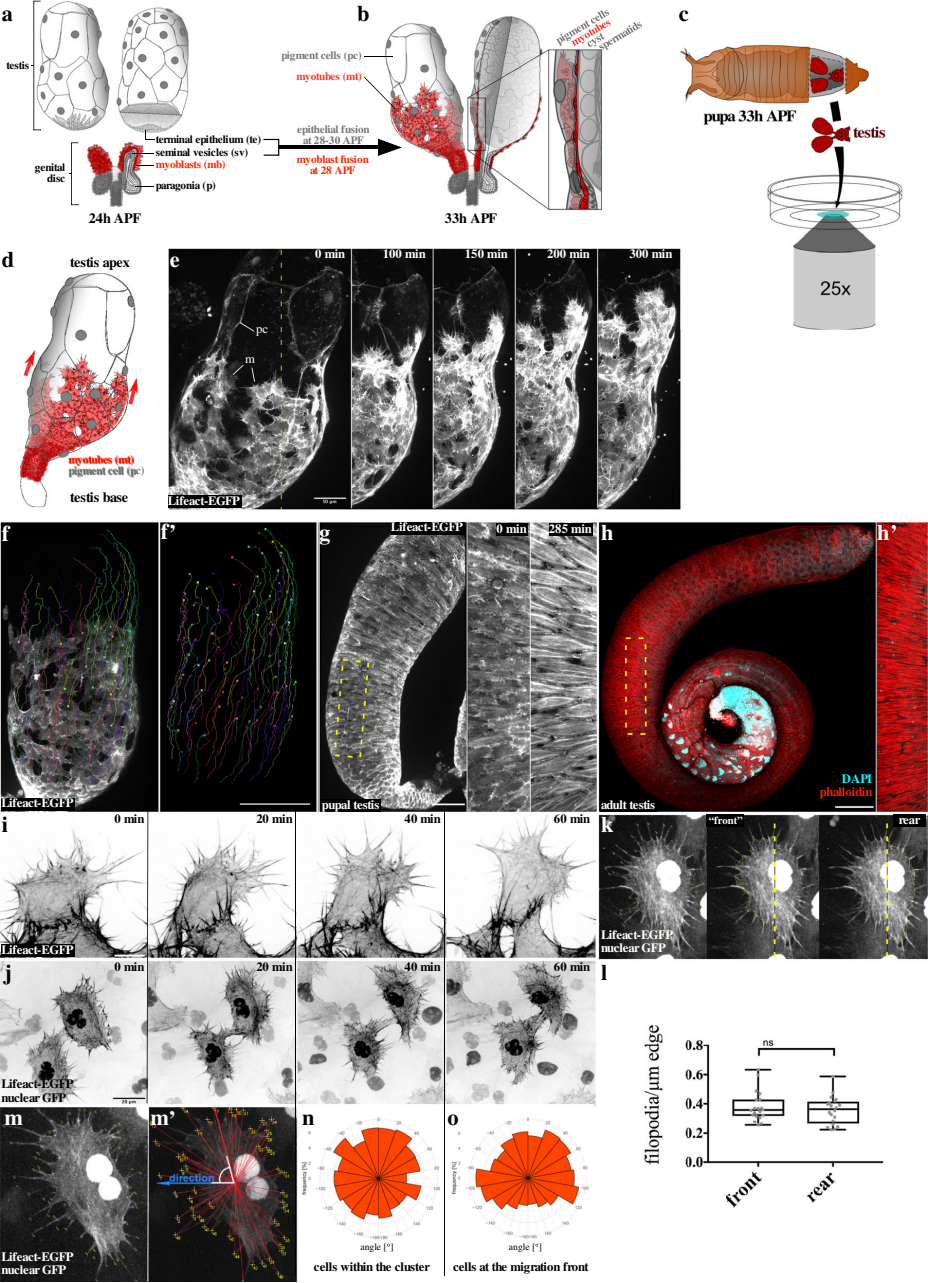
Myotubes form numerous filopodial membrane protrusions instead of lamellipodia and migrate collectively onto the testis. **a**, **b** Graphics of the *Drosophila* testis at 24 and 33 h after pupae formation (APF). **a** Myoblasts (mb, red), seminal vesicles (sv), and terminal epithelia (te) are indicated. **b** Myotubes (mt) start to migrate beneath the pigment cell layer (pc). **c** Schematic of the ex vivo technique. **d** Only one testis of the pair is depicted. **e** Wild-type testis. LifeAct-EGFP is expressed in migrating myotubes (m) and pigment cells (pc). The dashed line in 0 min represents the area depicted in 100–420 min. Scale bar, 50 µm. **f**, **f**’ Myotubes expressing LifeAct-EGFP were tracked using the Imaris software. Different colors represent individual cell tracks. **f** An overlay of microscopic data and track data are shown. **f**’ Only track data are shown. Scale bar, 100 µm. **e** Top view of a testis 46 h APF in 420 min ex vivo culture. *mef2-Gal4* drives UAS-LifeAct-EGFP expression. **g** Subsequent to migration, testis myotubes start to encircle the testis, generating ring muscles. The dashed line in h represents the area depicted in 0/285 min. Scale bar, 100 µm. **h** Adult testis with its typical coiled shape stained with phalloidin and DAPI. Due to constriction by building muscles in pupal development, the testis gained its typical coiled shape^30^ Scale bar, 100 µm; close-up in **h**’. **i** Myotubes at the front edge of the migrating sheet 60 min in ex vivo culture. Scale bar 10 µm. **j** Close-up of two myotubes during migration 60 min in ex vivo culture. *beatVC-Gal4* induces mosaic expression of UAS-LifeAct-EGFP and UAS-GFP-nls. Nuclei are marked by yellow asterisks. Scale bar, 20 µm. **k**, **l** Quantification of filopodia number per cell edge length as depicted in **k**. *N* = 20 cells. Boxplot center: median. Bounds: 25th and 75th percentiles. Whiskers: minimum/maximum values. *P ≤ 0.05, **P  ≤ 0.01, ***P  ≤ 0.001, ****P  ≤ 0.0001. Statistical testing: Mann–Whitney test (two tailed). Directionality of filopodia of cells was quantified by measuring the orientation angle as illustrated in **m** and **m**’. Quantification revealed no strong bias in filopodia direction neither of cells **n** within the myotube cluster nor **o** of cells at the migration front.

At 24 h after puparium formation (APF), both testes lay free in the body cavity (Fig. 1a). The genital disc provides the myoblasts and other somatic parts of the reproductive system such as the seminal vesicles^17,18^. Testes myoblasts adhere to the epithelium of the seminal vesicles (Fig. 1a, sv) and fuse to small syncytia shortly before the connection between seminal vesicles and terminal epithelia (Fig. 1a, te) has been formed (Fig. 1a, b)^19,20^. Between 28 and 30 h APF, this connection has been established (Fig. 1, see arrow between a and b). At 30 h APF, nascent myotubes (Fig. 1b, mt in red) start to migrate beneath the pigment cell layer (Fig. 1b, pc) to and along the testes toward the apical end (Fig. 1b)^21^. At 40 h APF, myotubes cover the whole pupal testis as a thin muscular sheet^22^.

To better understand how myotubes cover the testis, we established a protocol for ex vivo organ cultivation and long-term imaging (7 h) of isolated 33 h APF pupal testes (Fig. 1c). We used the muscle-specific *mef2*-Gal4 or the *heartless*-Gal4 (*htl*-Gal4) driver to express a UAS-LifeAct-EGFP transgene either in myotubes or in both, myotubes and pigment cells, respectively, (see Supplementary Fig. 2a). This method provides an excellent experimental system for studying the highly dynamic migratory cell behavior of myotubes and to visualize their actin-rich protrusions over several hours at high resolution. Spinning disc live-imaging microscopy of 33 h APF old testes onward revealed that myotubes migrate collectively on an ellipsoid surface constrained by the outermost layer of pigment cells and the basal membrane enclosing the inner cysts (Fig. 1d, e; Supplementary Movie M1). To better track the migratory behavior of individual cells within the cell cluster, we additionally labeled the cells by co-expression of the membrane marker mCD8-RFP enabling precise 4D (*xyz* and *t*) trajectory mapping using the Imaris software (Fig. 1f; Supplementary Movie M2). Since all mathematical directionality descriptors for 2D migration (biased angle, persistence angle, straightness, etc.) are based on Euclidean geometry, we had to transform our 3D(+time) datasets into corresponding 2D(+time) datasets for precise cell quantification. A simple *xy*-projection would neglect curvature and lead to wrong results. Preexisting tools using unwrapping algorithms and Riemannian manifold learning were not compatible with our system^23^. Instead of an unwrapping algorithm fit for every kind of surface, but with some restrictions in angle and distance accuracy, we developed a Mercator-projection-based process, which allows for high angle accuracy but neglects distances (illustrated in Supplementary Fig. 3b–f).

Dissecting the cell trajectories of wild-type myotubes revealed a directional cell behavior with maximal cell movement into the base-apex direction with a speed about 0.37 µm/min over a distance of about 130 µm (Fig. 1f’, Supplementary Fig. 2g; Supplementary Movie M2). Once myotubes reached the testis apex they started to elongate and form large actin bundles that aligned perpendicular to the pupal testis surface (Fig. 1g, Supplementary Movie M1, middle). After completing pupal development, myotubes form a densely packed muscle sheath surrounding the elongated, tubular adult testis (Fig. 1h, h’).

### Myotube migration depends on formin-dependent filopodial membrane protrusions

Strikingly, migrating myotubes largely lacked lamellipodial protrusions, but instead formed numerous filopodia-like protrusions (from here on referred to as filopodia; Fig. 1i; Supplementary Movie M3). Expression of LifeAct-EGFP together with a nuclear targeted EGFP transgene in myotubes in a mosaic-like fashion further showed that myotubes also formed prominent filopodial protrusion between neighboring cells (Fig. 1j; Supplementary Movie M4). To better characterize the distribution of filopodia in these cells, we quantified the directionality of filopodia of cells by measuring the orientation angle as illustrated in Fig. 1m, m’. This analysis revealed no strong bias in filopodia generation or directionality in cells within the cluster (Fig. 1n) and surprisingly also at the front edge of the cluster (Fig. 1o). To statistically analyze this, we differentiate the filopodia (in cells at the front edge) in those which are assembled at the cell front (pointing to the testis apex) and those at the rear (pointing to the testis base; Fig. 1k, l). To account for irregular cell shapes, we calculated the density (number/µm) by measuring edge length. There was no significant difference in filopodia density between front and rear. Thus, the directionality of collective myotube migration cannot be simply predicted by filopodia number or direction.

**Fig. 2:**
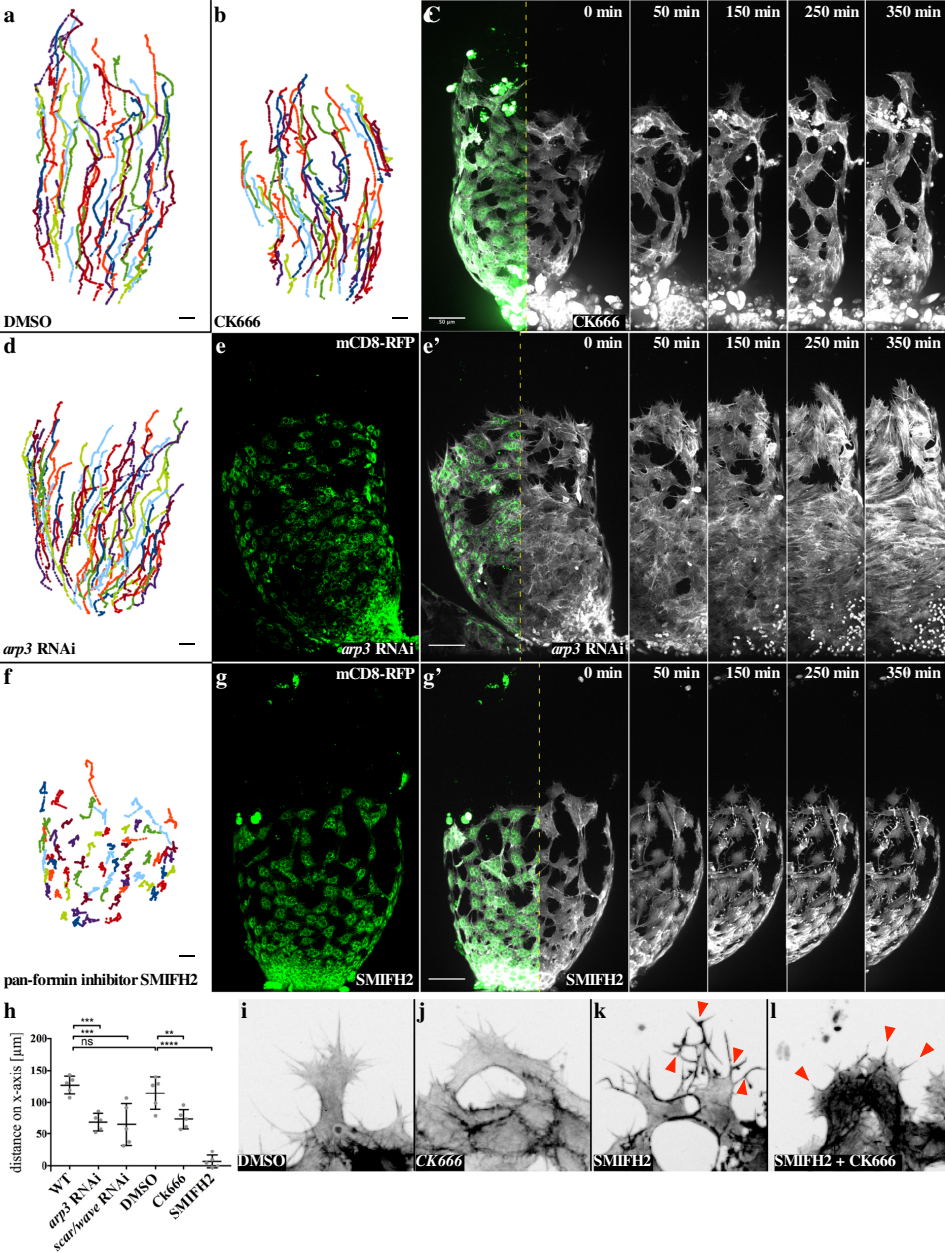
Formins are essential in myotube collective migration and filopodia dynamics, but not in the Arp2/3 complex. **a** Migration tracks of testis myotubes 33 h APF in 420 min ex vivo culture, treated with DMSO as a control. Different colors represent individual cell tracks. **b**, **c** CK666 (100 µM) treatment of a testis 33 h APF in 420 min ex vivo culture. Upon Arp2/3 complex activity inhibition, migration is reduced. *mef2-Gal4* drives UAS-LifeAct-EGFP and UAS-mCD8-RFP expression. **b** Migration tracks of testis myotubes upon CK666 treatment. Different colors represent individual cell tracks. **b** Life imaging micrographs. mCD8-RFP in green and LifeAct-EGFP in white. The dashed line in **c** represents the area depicted in 0–420 min. Scale bar, 50 µm. **d**, **e** Migration is also mildly reduced by *arp3* RNAi. *mef2-Gal4* drives UAS-LifeAct-EGFP, UAS-mCD8-RFP, and the RNAi construct *UAS-arp3*^*KK102278*^ (Vienna v108951). **d** Migration tracks of testis myotubes upon *arp3* RNAi. Different colors represent individual cell tracks. **e**, **e**’ Life imaging micrograph. mCD8-RFP (green) is depicted in **e**. (Note: mononucleated myotubes marked by co-expression of the mCD8-RFP marker are excluded from the nuclei.) Overlay with LifeAct-EGFP (white) in **e**’. The dashed line in **e**’ represents the area depicted in 0–350 min. Scale bar, 50 µm. **f**, **g** Upon formin suppression through SMIFH2 (10 µM) treatment, migration is completely disrupted. *mef2-Gal4* drives UAS-LifeAct-EGFP and UAS-mcd8-RFP expression. **f** Migration tracks of testis myotubes upon SMIFH2 treatment. Different colors represent individual cell tracks. **g**, **g**’ Life imaging micrographs. mCD8-RFP (green) is depicted in **g**. Overlay with LifeAct-EGFP (white) in **g**’. The dashed line in **g**’ represents the area depicted in 0–350 min. Scale bar, 50 µm. **h** Quantification total migration distance along *x*-axis. *N* = 5 testes. Data are presented as mean values ± SD. *P ≤ 0.05, **P  ≤ 0.01, ***P  ≤ 0.001, ****P  ≤ 0.0001. Statistical testing: one-way ANOVA. **i**–**k** Close-ups of front-row myotubes upon different treatments. **i** Upon DMSO treatment, cell morphology and filopodia composition were not affected. **j** Arp2/3 suppression by CK666 treatment leads to mild defects. No branched filopodia are built, the overall morphology is unaffected. **k** Formin suppression by SMIFH treatment leads to strong morphological defects. Cells are contracted, filopodia generate more branches. **l** CK666 in addition to SMIFH2 co-treatment leads to a loss of branched filopodia. Cells are contracted even stronger.

We next determined how central actin nucleators such as formins and the Arp2/3 complex contribute to filopodia formation and myotube migration. Treatment with the specific Arp2/3 inhibitor CK666^24^ did not strongly affect the overall cell cluster morphology compared to control cells incubated with DMSO (Fig. 2a–c; Supplementary Movie M5). Likewise, cells depleted of the *arp3* subunit or *wave* by RNA interference (RNAi) showed moderate changes in cell morphology despite prominent fusion defects (see mononucleated myotubes marked by co-expression of the mCD8-RFP marker excluded from the nuclei in Fig. 2e; Supplementary Movie M6). Similar to CK666 treatment, *arp3* and *wave* depleted cells were still able to migrate persistently in a directed fashion (Fig. 2b–e’; Supplementary Fig. 2h. However, cells depleted of the *arp3* subunit or treated with CK666 showed a significantly reduced migration speed and distance along the *x*-axis (compare migratory tracks in Fig. 2a, b, d; quantification in Fig. 2h, Supplementary Fig. 2g). Thus, the Arp2/3-WRC pathway promotes motility, but seems to be dispensable for directed migration of myotubes.

**Fig. 3:**
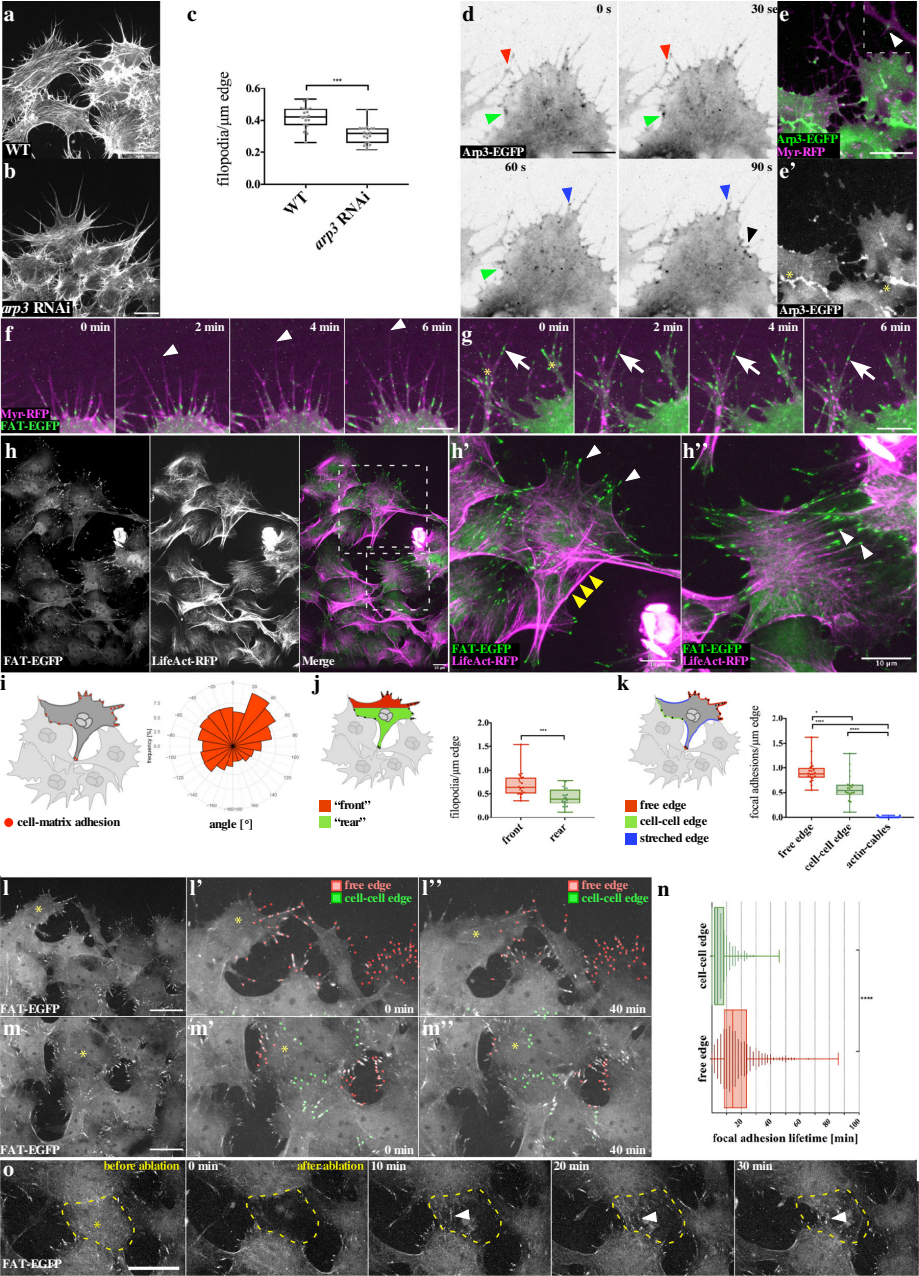
Migrating myotubes form stable cell–matrix adhesions at their free edge and adherens junctions at their cell–cell edge. **a** Wild-type and **b** *arp3* knockdown myotubes. Scale bar, 10 µm. **c** Quantification of filopodia number per cell edge length. **d** Still images of a front-row myotube expressing Arp3-EGFP. Arrowheads mark positions of Arp3 at filopodial branch points. Scale bar, 10 µm. **e** Still images of a front-row myotube coexpressing Arp3-EGFP and Myr-RFP. The arrowhead marks a position of Arp3. Scale bar, 10 µm. **f**, **g** Close-up of myotubes at the front edge of the migrating sheet, 6 min in ex vivo culture. Cell–matrix adhesions (green) are found in a beaded string-like manner along filopodia. Scale bar: 10 µm**. g** Some filopodia build branches. UAS-FAT-EGFP, scale bar: 10 µm. **h** Dashed lines represent the area magnified in **h’** and **h”**. **h’** Matrix adhesions are found at filopodia tips (white arrowheads). Actin cables are marked by yellow arrowheads. **h”** Cell–matrix adhesions colocalize with actin fibers (white arrowheads). **h’**, **h”** Scale bar, 10 µm. **i**–**k**. Boxplot center: median. Bounds: 25th and 75th percentiles. Whiskers: minimum/maximum values. *P ≤ 0.05, **P  ≤ 0.01, ***P  ≤ 0.001, ****P  ≤ 0.0001. Quantification revealed **i** a significant bias in directionality of cell–matrix adhesions, and **j** an increased number of cell–matrix adhesions at the cell front, *N* = 20 cells. Mann–Whitney test (two tailed) and **k** an increased number of cell–matrix adhesions at the free edge compared to cell–cell edges. *N* = 20 cells. Kruskal–Wallis test. **l**, **m** Cell–matrix adhesions during migration. Quantified adhesions at the free edge are depicted in red and at the cell–cell edge in green. Scale bar: 10 µm. **l** Front edge of the migrating sheet. **l’** Magnification at 0 min. **j”** Magnification at 40 min. **m** Following cells in the same sheet as in H. **m’** Magnification at 0 min. **m”** Magnification at 40 min. **n** Quantification revealed that cell–matrix adhesions longevity is significantly higher at the free edge” compared to the cell–cell-edge”. *N* = 980 matrix adhesions in three testes. Mann–Whitney test (two tailed). **o** Cell–matrix adhesions before and after laser ablation. After laser ablation, new cell–matrix adhesion formed along the free edge (arrowhead). Scale bar: 10 µm.

By contrast, treatment with the pan-formin small-molecule inhibitor SMIFH2^25^ strongly affected cell morphology and completely disrupted collective myotube migration (Fig. 2f, g, g’; Supplementary Movie M7; quantification in Fig. 2h, g). Compared to CK666 treatment, cells treated with SMIFH2 showed a prominent reduced number of dynamic, but instead highly branched filopodia-like protrusions (Fig. 2k; Supplementary Movie M7). Interestingly, cells co-treated with CK666 and SMIFH2 completely lacked these branched filopodial protrusions suggesting that their formation or branching depends on a still prominent Arp2/3 complex activity in SMIFH2 treated cells (Fig. 2l). Supporting this notion, cells only depleted for Arp3 showed a reduction in filopodia branches resulting in a significant reduction of protrusions (Fig. 3a, b; quantification in c). Consistently, an Arp3-EGFP transgene localized close to newly forming branches as we recently found in dendrite branchlet formation of *Drosophila* larval sensory neurons (arrowheads in Fig. 3d, e)^26^. Interestingly, we also found a strong accumulation of the Arp3-EGFP at cell–cell contacts, an observation made in different cell systems (asterisks in Fig. 3e’).

Taken together, these findings suggest that Arp2/3 activity is required in filopodia branching, whereas the activity of formins is essential to generate filopodial protrusions. RNAi-mediated suppression of single *Drosophila* formins did not result in prominent protrusion or migration defects (see Supplementary Table suggesting potential redundant and synergistic functions of different formins in protrusion formation.

### Migrating myotubes preferentially form more stable focal adhesions at their free edge

It is generally believed that filopodia may promote mesenchymal migration by promoting cell–matrix adhesiveness at the leading edge to stabilize the advancing lamellipodium^27^. Migrating myotubes lack lamellipodia, but instead filopodia appear to be critical for myotube migration as inhibition of their formation by interfering with formin function results in a complete loss of migration. Expression of a cell–matrix adhesion targeting reporter (FAT-EGFP^28^) revealed that migrating myotubes indeed formed numerous cell–matrix anchorage sites at the base, along the shaft, and at the tip of filopodia (Fig. 3f; Supplementary Movie M8). Multiple cell–matrix adhesions were built in a single filopodium, giving them a beaded appearance (Fig. 3g; Supplementary Movie M8). Cell–matrix adhesions formed along filopodia shafts subsequently seemed to move rearward, along a retrograde flow of bundled actin filaments, eventually getting disassembled in the outer rim of the cell body (Fig. 3g; Supplementary Movie M8). Co-expression with a LifeAct-RFP reporter marked especially thicker actin bundles attached to large, more elongated cell–matrix adhesion structures that shows a more classical appearance of matrix adhesions found in lamellipodia (Fig. 3h–h”).

Remarkably, the number of cell–matrix adhesions within single cells at the front edge of the cluster correlates with the presumed direction of migration toward the testis tip (Fig. 3k). Cells formed an increased number of cell–matrix adhesions at the migrating front (pointing to the testis apex; Fig. 3i) when compared to the rear. An even more pronounced difference becomes apparent, when instead of comparing front and rear, cell–matrix adhesions are divided into those build at the free edge (excluding free edge regions comprising actin cables marked in blue) versus the cell–cell edge as illustrated in Fig. 3j, k. Quantitative analysis of matrix adhesion dynamics further showed that cell–matrix contacts formed at free edges showed significantly increased lifetime compared to those close to cell–cell contacts (Fig. 3l–l”; Supplementary Movie M9; quantification in Fig. 3n). This asymmetric distribution of cell–matrix adhesion implies that polarization along the cell edge of myotubes does not require specialized leader cells, as observed in endothelial cells or border cell migration^29^. It rather appears to be a response on exhibiting free edge and potentially can occur in every cell within the cluster. Consistently, an increase of free edges within the cell cluster was accompanied with the formation of new matrix adhesions as ablation experiments showed. Myotubes immediately migrated when exposed to an empty space and filled the gaps within laser-induced wounds (Fig. 3o; Supplementary Movie M10).

### Reduced N-cadherin expression promotes single-cell migration at the expense of collective directionality

**Fig. 4:**
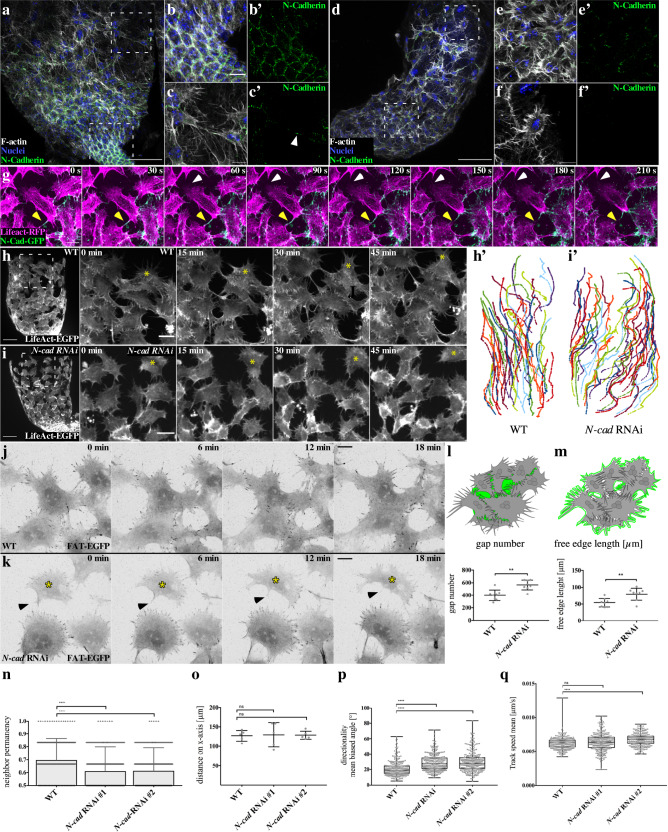
Reduced N-cadherin expression increases free edge, promoting cell-independent behavior at the expense of collective directionality. **a**–**f** Wild-type testis stained for N-cadherin, F-Actin and DAPI. **a** Overview of the testis base. Areas marked with dashed lines are magnified in **b**, **c**, **e**, and **f**, respectively. Scale bar: 50 µm. **b** On the genital disc myotubes appear epithelial. **c**, **c**’ At the front edge of the migrating sheet, N-cadherin localized in foci along filopodia (white arrowhead). **d**, **e**, **e**’ The same is true for cells within the sheet. **f**, **f**’ In rare cases, completely isolated cells without N-cadherin staining could be observed. **b**, **c**, **e**, **f** Scale bar: 10 µm. **g** Still images of myotubes expressing N-cad-EGFP. Arrowheads mark positions of N-cad-EGFP enriched cell–cell junctions. Scale bar, 10 µm. **h** Wild-type (WT) testis 33 h APF in ex vivo culture. Overview at *t* = 0 min at the left side. Scale bar: 50 µm. Time steps from 0 to 45 min in ex vivo culture. Scale bar: 20 µm. **h’** Tracks of wild-type and **i** N-cadherin knockdown myotubes. Isolated myotubes are marked by yellow asterisk (*t* = 0 min, 45–60 min). Different colors represent individual cell tracks. **i’** Tracks upon knock down of N-cadherin. Different colors represent individual cell tracks. **j** Wild-type and **k** N-cad RNAi myotubes. Scale bar: 20 µm. **l**–**o** Data are presented as mean values ± SD. *P ≤ 0.05, **P  ≤ 0.01, ***P  ≤ 0.001, ****P  ≤ 0.0001. **l**, **m** Graphical representation of the values is depicted. **l** The number of gaps between cells is significantly increased. *N* = 8 testes. Unpaired *t* test (two tailed). **m** As proxy for free edge, we used the perimeter of the white-to-black edge. Cell-free edge is significantly increased in *N-cad* RNAi animals. *N* = 8 testes. Kruskal–Wallis test. **n** Neighbor permanency is significantly reduced in N-cadherin knock down. **o** Quantification of the migration distance. *N* = 5 testes. One-way ANOVA. **p**, **q** Boxplot center: median. Bounds: 25th and 75th percentiles. Whiskers: minimum/maximum values. **n**, **p**, **q** *N* = 5 testes with 289 cells (WT), 304 cells (RNAi #1), and 291 cells (RNAi #2). Statistical testing: Kruskal–Wallis test. **p** Biased angle in regard to the testis axis was measured. **q** Quantification of track speed mean in µm/s.

Reduced cell–matrix adhesion density of myotubes in contact might be due to an enhanced disassembly of cell–matrix complexes at cell–cell contacts as previous reported for neural cells undergoing CIL^13^. Migratory myotubes predominantly express N-cadherin as a key adhesion molecule of cell–cell contacts^30^, which is essential in early *Drosophila* embryogenesis^31^. N-cadherin was not only found along adjacent membranes of myotube sheets at the testis base (Fig. 4a, b, b’), but were also highly enriched along the bridges of interdigitating filopodia (Fig. 4c, c’, e, e’). In contrast, single myotubes without any cell neighbor that were rarely observed (Fig. 4f, f’) completely lacked N-cadherin clusters at their free edge filopodia. Live imaging of migrating myotubes expressing a N-cad-EGFP transgene confirmed a highly dynamic accumulation at cell–cell contacts and along filopodia forming initial contacts between neighboring cells (Fig. 4g; see also Supplementary Movie M11).

To further test the importance of N-cadherin-dependent cell–cell contacts in controlling the collective behavior of myotubes, we used an RNAi approach to downregulate N-cadherin expression in myotubes by using the *mef*2-Gal4 driver (Fig. 4h, i; Supplementary Movie M12). Expression of two different RNAi transgenes efficiently downregulates N-cadherin protein level as shown by immunostainings of adult testes (Supplementary Fig. 2h, i, quantification in j). Myotubes depleted for N-cadherin are still able to migrate, and even change more frequently their relative positions with each other within the moving cluster (Fig. 4h, i; Supplementary Movie M12). Expression of different *N-cad* RNAi transgenes resulted in an obvious increase of free cell edges with prominent cell–matrix adhesions (Fig. 4j, k; Supplementary Movie M13) and increased gaps between migrating myotubes (Fig. 4l, m) in a dosage-dependent manner, but did not affect the cell number or cell size (Supplementary Fig. 2b–e). Consistently, suppression of N-cadherin led to a significantly decreased neighbor permanency suggesting that indeed a reduced N-cadherin function weakened cell–cell adhesions (Fig. 4n). Quantitative analysis of the migration pattern of individual cells further revealed prominent changes of the migratory behavior. Overall, the total migration distance along the *x*-axis was not affected indicating that N-cadherin-depleted cells migrate as far as wild-type cells (Fig. 4o; Supplementary Movie M12). However, N-cadherin-depleted cells migrate significantly less directional but faster compared to wild-type cells (Fig. 4p, q). Thus, myotubes did not display a leader–follower cell dynamics, in which leader cells drag inherently passive followers cells by means of strong cell–cell cadherin contacts. By contrast, N-cadherin-mediated cell–cell contacts seem to be required for the directionally coordinated migratory behavior of myotubes.

### Migrating myotubes need cell–cell contact to achieve directionality

**Fig. 5:**
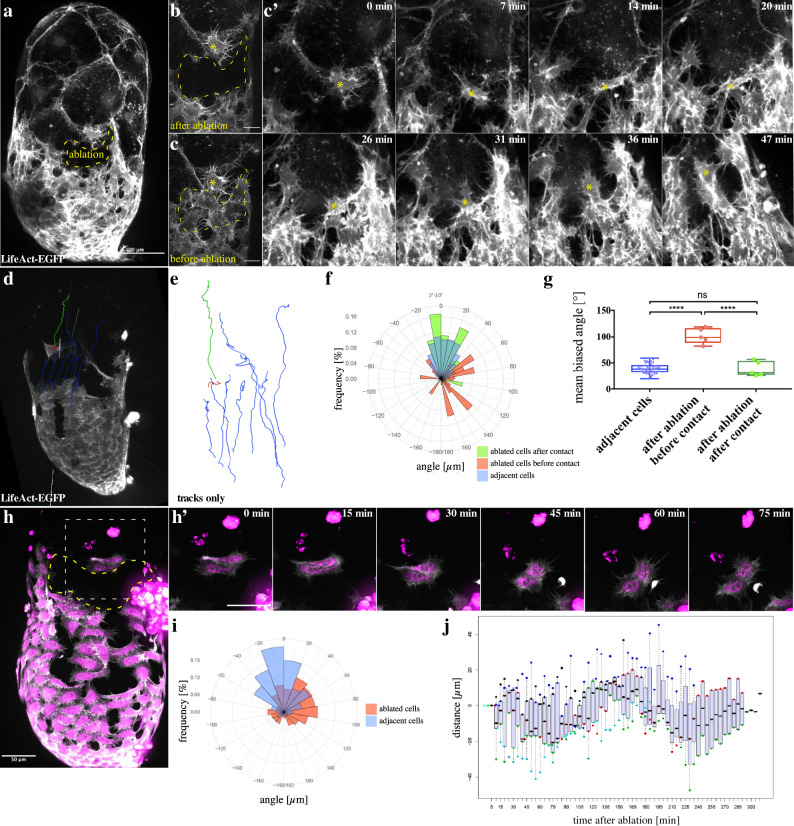
Migrating myotubes need cell–cell contact to achieve directionality. **a–g** Isolation of a single nascent myotube by laser ablation. **a** Overview of a testis after laser ablation (33 h APF). *htl-Gal4* drives UAS-LifeAct-EGFP expression. Scale bar, 100 µm. **b** Close-up on the ablation site. **c** Same site as in **b**, before ablation. Scale bar in **c** and **c’**: 20 µm. The dashed line represents the area affected by laser ablation. **c’** Behavior of the isolated cell from **b** after ablation. The isolated cell (yellow asterisk) shows no forward motion if it has no contact to adjacent cells (upper row). After contact is established, it moves along in the migrating sheet (bottom row). **d**, **e** To quantify the directionality of the isolated cell, cell motion was tracked using the Imaris software. The isolated cell before contacting to the migrating sheet is depicted in red, after contacting it is depicted in green. As a control, adjacent cells were tracked. They are showed in blue. **f**, **g** As a measurement tool, we used the biased angle to *x*-axis. The mean angle (0–180°) of every track is blotted. When isolated, cells lose their directionality, but regain it after establishing contact to adjacent cells. *N* = 5 testes with 38 cells (adjacent cells), 5 cells (after ablation, before contact), and 5 cells (after ablation, after contact). Boxplot center: median. Bounds: 25th and 75th percentiles. Whiskers: minimum/maximum values. *P ≤ 0.05, **P  ≤ 0.01, ***P  ≤ 0.001, ****P  ≤ 0.0001. Statistical testing: one-way ANOVA. The color code is the same as in **e**, **f** *N* = 5 testes. **h**–**j** Isolation of two adjacent myotubes by laser ablation. **h** Overview of a testis after laser ablation (33 h APF). *htl-Gal4* drives LifeAct-EGFP (gray) and Myr-RFP (magenta) expression. The dashed line represents the area affected by laser ablation. Scale bar, 100 µm. **h’** Behavior of the two isolated myotubes from **h** after ablation. Scale bar in **c** and **c’**: 20 µm. **i** Rose plot shows the distribution of the biased angle to *x*-axis. **j** Measurement of the distance between two myotubes over time.

To further test whether myotubes require cell–cell contacts for their directional cell migration, we performed laser ablation experiments. Isolation of single myotubes by laser ablation of the adjacent neighboring cells on the testis created a situation, in which a cell is surrounded by free edge. After the ablation, the isolated cells immediately ceased directional migratory behavior and cells formed numerous filopodial protrusions pointing in all directions (Fig. 5a–c; Supplementary Movie M14). Once those cells got in close contact to adjacent cells, they started to migrate forward along those migratory sheets as a collective (Fig. 5d, e; Supplementary Movie M14). Single-cell tracking before and after cell–cell contact confirmed a contact-dependent migratory cell behavior of myotubes, a phenomenon that is reminiscent of CIL (Fig. 5e–g). Remarkably, such a contact-stimulated migratory behavior could not be observed between two individual cells, which were still connected by cell–cell junctions but isolated from remaining cell cluster by laser ablation (Fig. 5h; Supplementary Movie M15). Cell pairs neither migrated away from each other nor became polarized pointing protrusions into opposite directions, but instead always stuck together with constant contact distance over time (Fig. 5i, j; Supplementary Movie M15).

### Rac2 and Cdc42 functions play important roles in myotube migration shaping testis morphology

Cell adhesions are not only required to mechanically couple cells within the cluster, but also to link adhesion complexes to the actin cytoskeleton controlling the protrusion dynamics and directionality^32^. Rho GTPases are critical molecular players that regulate adhesions and motility during single and collective cell migration^33,34^.

To identify such key players contributing to myotube migration we used an RNAi approach to screen numerous candidate genes (see Supplementary Table). Defects in testis myotube migration during pupal metamorphosis can be identified by a prominent disturbed morphology of adult testis (Supplementary Fig. 1a–l)^30^. The adult testis is a pair of thin tubules of 2.5 coils and ∼2 mm in length surrounded by a sheath of multinuclear smooth-like muscles^19,30^. Defective N-cadherin-mediated cell–cell adhesion resulted characteristic holes in the muscle sheet^19^, where myotubes were not properly attached to one another (Supplementary Fig. 1a, c, e). In contrast, defects in myotube migration resulted in an abnormal testis morphology with reduced coils and bulky tips (Supplementary Fig. 1a, f–k). Depending on the phenotypic strength the muscle sheath only partially or completely failed to cover the entire testis resulting into strong elongation/coiling defects (Supplementary Fig. 1a). Strong abnormalities were observed following RNAi-mediated suppression of Cdc42 and Rac2 functions, one of the two very similar *rac* genes in *Drosophila*^35^. In both cases, the adult testes were smaller than in the wild type with reduced coils and bulky tips (Supplementary Fig. 1f, g). The muscle sheath either did not cover the entire testes with numerous large holes. In comparison, suppression of Arp2/3 complex subunits and single subunits of the WRC^36^ such as WAVE and the Rac-effector Sra-1, resulted into more moderate morphological defects compared to *rac2* or *cdc42* depletion. Adult testes deficient for Arp3, WAVE. and Sra-1 still had about 1.5–2 coils, however many myotubes also did not reach the testis apex resulting into bulky tips (Supplementary Fig. 1h–j).

### Rac2 and Cdc42 are required for myotube migration by differentially regulating cell–matrix adhesions

**Fig. 6:**
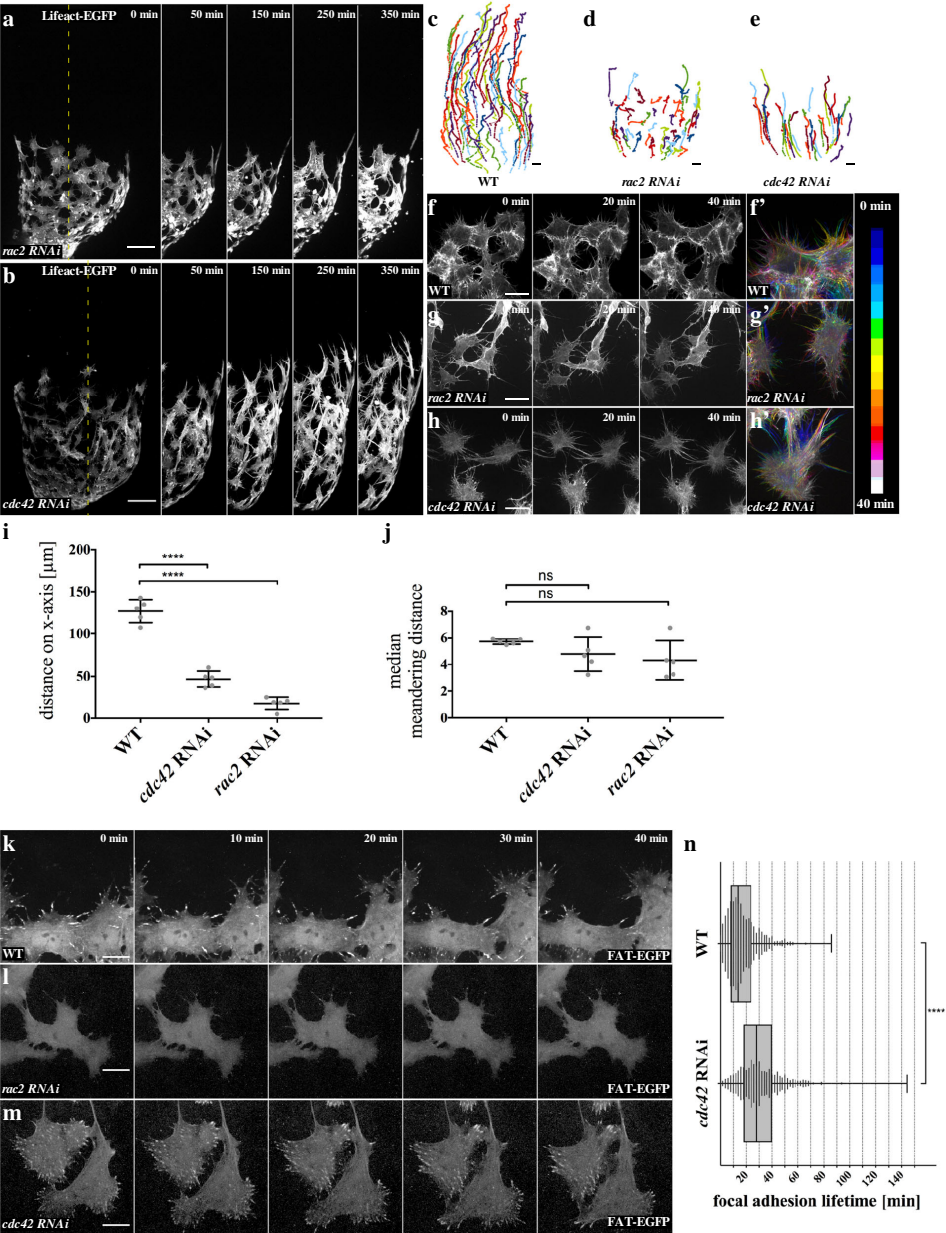
Rac2 and Cdc42 regulate filopodia matrix adhesion to enable myotube collective migration. **a**, **c**, **i**, **j** *rac2* knock down was induced by expression of the *UAS-rac2*^*NIG.8556R*^ RNAi transgene together with UAS-LifeAct-EGFP, using *mef2-Gal4*. **a** *rac2* knock down in myotubes on testis 33 h APF in ex vivo culture. Tracks are depicted in **d**. Different colors represent individual cell tracks. The dashed line in 0 min represents the area depicted in 50–420 min. Scale bar: 50 µm. **b**, **d**, **i**, **j** *cdc42* knock down was induced by expression of the *UAS-cdc42*^*TRiP.JF02855*^ (#1) or the *UAS-cdc42*^*KK108698*^ (#2) RNAi transgenes, together with UAS-LifeAct-EGFP, using *mef2-Gal4*. **b** *cdc42* knock down in myotubes on testis 33 h APF in ex vivo culture. Tracks are depicted in **e**. Different colors represent individual cell tracks. The dashed line in 0 min represents the area depicted in 50–420 min. Scale bar: 50 µm. **f**–**h** Close-up of myotubes 33 h APF in ex vivo culture with corresponding color-coded projection in **f’-h’** Scale bar: 10 µm. **f**, **f’** Wild-type (WT) myotubes. **g**, **g’** *rac2* RNAi causes a fast assembly and disassembly of filopodia. **h**, **h”** *cdc42* RNAi leads to very stable filopodia in comparison to wt. Filopodia are prolonged, even between nascent myotubes, rendering close cell–cell contact harder to achieve, thus the entire sheet appears less dense as in WT. **i** Quantification of migration distance on *x*-axis (compare to Fig. 3j). **j** Quantification of median meandering distance. **i**, **j**, **n** *P ≤ 0.05, **P  ≤ 0.01, ***P  ≤ 0.001, ****P  ≤ 0.0001. **i, j** Data are presented as mean values ± SD. *N* = 5 testes. Statistical testing: one-way ANOVA. **k** Matrix adhesions in myotubes during migration. Scale bar: 10 µm. **l** Matrix adhesions are completely lost upon *rac2* suppression by RNAi. **m** Matrix adhesions remain much longer upon *cdc42* reduction, even reaching the trailing end of a migrating cell. **n** Quantification of matrix adhesion lifetime. *N* = 980 matrix adhesions in three testes (WT) and *N* = 665 matrix adhesions in three testes (*cdc42* RNAi). Boxplot center: median. Bounds: 25th and 75th percentiles. Whiskers: minimum/maximum values. Statistical testing: Mann–Whitney test (two tailed).

Compared to suppression of the Arp2/3-WRC pathway, knock down of Rac2 functions led to stronger defects in membrane protrusions and cell migration suggesting that Rac2 might have additional roles in myotube migration (Fig. 6a, d; compare quantification in Supplementary Fig. 2g). *rac2*-depleted cells showed a severely changed cell morphology with thinner and highly dynamic filopodial protrusions. These filopodia were unable to adhere stably (Fig. 6g, g’; Supplementary Movies M16 and M17). Supporting this notion, live-cell imaging of *rac2* knockdown cells using the FAT-EGFP reporter revealed a prominent loss of cell–matrix adhesion contacts (Fig. 6l, Supplementary Movie M18). Since *mef2*-Gal4 driven FAT-EGFP is still normally enriched in integrin-dependent adhesion structures such as muscle attachment sites of the larval body wall musculature, a general impact of Rac2 function on matrix adhesion can be excluded (Supplementary Fig. 1m, n).

Suppression of Cdc42 function also severely impaired migration speed resulting in a strongly reduced migration distance on the *x*-axis (Fig. 6b, e; Supplementary Movie M15; compare quantification in Supplementary Fig. 2g). However, compared to *rac2*-depleted cells, *cdc42*-deficient myotubes showed an increase of thin and prolonged filopodia (Fig. 6g, g’; Supplementary Movies M17 and M19). Overall, the *cdc42*-depleted myotubes showed an elongated cell shape with numerous gaps between adjacent cells. Live-cell imaging of *cdc42* knockdown cells using the FAT-EGFP reporter revealed a significantly increased lifetime of cell–matrix adhesions (Fig. 6m; quantification in n; Supplementary Movie M18). Compared to wild-type cells, the cell–matrix adhesions remained much longer, even when they reached the trailing end of a migrating cell (Supplementary Movie M18). In summary, Rac2 and Cdc42 are both required for myotube migration, but appear to differentially regulate cell–matrix adhesions.

### Activated Rho1 is not enriched at cell–cell contacts

The activity of Rho1^37,38^, the *Drosophila* homolog of RhoA, appears to be as essential for myotube migration as Cdc42 and Rac2. RNAi-mediated suppression of Rho1 but not RhoL activity in myotubes indeed resulted in strong morphological defects of the testes, and even under low RNAi transgene expression (using *lbe*-Gal4 driver) *rho1* depleted myotubes showed strong migration defects (see Supplementary Fig. 1k, Supplementary Table). Suppression of the same RNAi transgenes using the mef4-Gal4 driver resulted into an early pupal lethality (Supplementary Table).

**Fig. 7:**
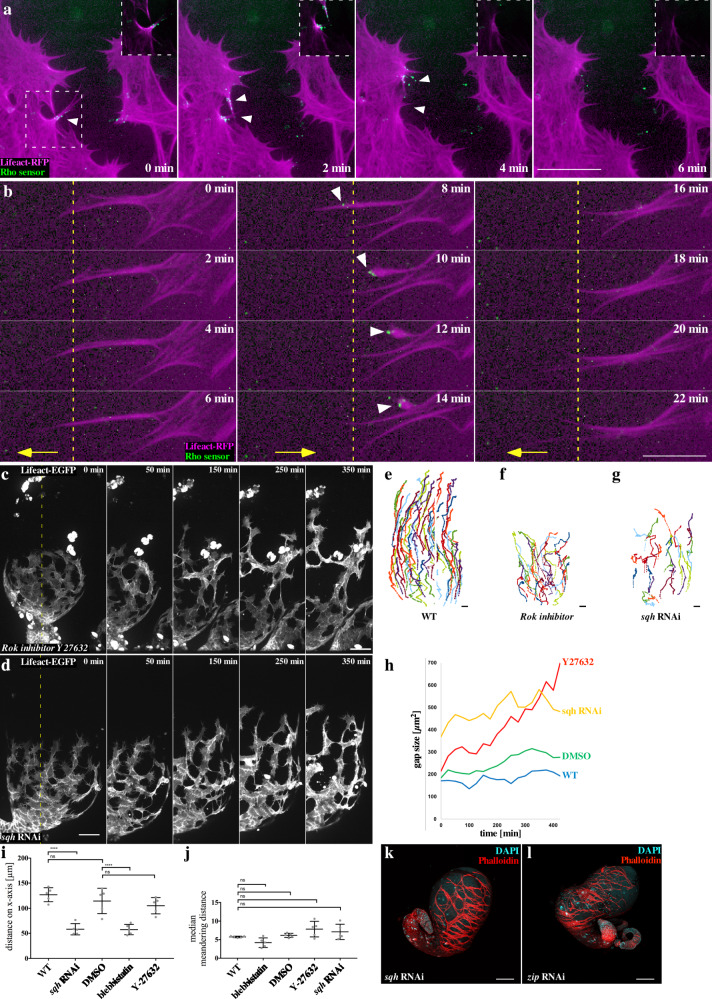
Rho/Rok-driven actomyosin contractility is essential for myotube migration. **a, b** Close-ups of myotubes at the front edge of the migrating sheet 30 min in ex vivo culture. *mef2-Gal4* drives UAS-LifeAct-RFP and the Rho1 sensor Anillin-RBD-EGFP. UAS-LifeAct-RFP is enriched along the membrane in actin cables. To depict all actin structures, gamma was set on 0.09. In the boxes in the upper right corner, details with gamma = 1 are depicted. **a** Rho1 Sensor activity is found in free edge filopodia (white arrowheads, as LifeAct-RFP rapidly bleached out in filopodia tips, EGFP signal appears partially outside the cell). When analyzed with gamma = 1, it becomes clear that Rho1 sensor is only present at parts of the edge containing actin cables. After the Rho signal appears, the corresponding part of the cell retracts, and the Rho1 signal immediately disappears. During retraction, the LifeAct-RFP signal at the retractive site goes back to normal intensity. **b** Rho1 sensor activity does not seem to mark rear polarity. Filopodia can protrude (left column, yellow line, then activate RhoA and retract (middle column, yellow line). Subsequently, neighboring filopodia can elongate again (middle and left column, yellow line). **c** Myotubes expressing LifeAct-EGFP treated with the Rok inhibitor Y-27632. **d** *sqh* knock down was induced by expression of the *UAS-sqh* RNAi transgene. Myotube cell cluster were still able migrate with reduced speed and become strongly elongated with long interconnecting cell processes. The dashed line in 0 min represents the area depicted in 50–420 min. Scale bar: 50 µm. Tracks are depicted in **e** wild type (WT), **f** Y-27632 treatment, and **g** *sqh* knock down. Different colors represent individual cell tracks. **h** Measurement of the gap size within cell cluster over time. **i** Quantification of migration distance on *x*-axis. **j** Quantification of the median meandering distance. **i**, **j** *N* = 5 testes. Data are presented as mean values ± SD. *P ≤ 0.05, **P  ≤ 0.01, ***P  ≤ 0.001, ****P  ≤ 0.0001. Statistical testing: ordinary one-way ANOVA. Confocal images of adult testes expressing **k** a *sqh* RNAi transgene and **l** a *zip* RNAi transgene under the *mef2-Gal4* driver, the muscle sheet is stained with phalloidin (red) and nuclei are stained with DAPI (cyan). Scale bar: 100 µm.

Different from neural crest cells undergoing CIL, activated Rho1 was not enriched at cell–cell contacts between myotubes (Fig. 7a). Live imaging of migrating myotubes coexpressing a Rho1-GTP biosensor or Anillin Rho-binding domain fused to GFP (Anil.RBD-GFP^39^ and a LifeAct-RFP transgene uncovered highly dynamic, local pulses of Rho1 activity along retracting filopodial protrusions at free edges (Fig. 7a, b; Supplementary Movie M20). Rho1 activation appeared to be synchronous with backward movement of retracting filopodial protrusions (Fig. 7b; Supplementary Movie M20). Once a protrusion has been completely retracted, activated Rho1 disappeared. Remarkably, retracting protrusions were often followed by new forward-directed protrusions at the same region without any Rho1 signal (Fig. 7b, Supplementary Movie M20). Thus, migrating myotubes are not simply polarized along a front-rear axis.

### Myotube migration requires Rok-dependent actomyosin contractility

Rho1 is known to control myosin II-dependent contraction through the protein kinase Rok shaping cells into tissue in a large variety of morphogenetic events during development^40,41^. To test whether Rok-dependent actomyosin-mediated contractility is required for myotube collective migration, we first inhibited contractility by treating ex vivo cultured pupal testes with the specific Rok inhibitor Y-27632^42^ and with blebbistatin^43^ or rather its photostable derivate para-nitro-blebbistatin^44^, which targets the action of the myosin II (Fig. 7c, Supplementary Movie M21). Compared to control cells treated with DMSO, we found similar striking changes in cell morphology in a time-dependent manner that eventually disturb myotube cell migration (Fig. 7c, e, f; Supplementary Movie M21). Following treatment with Y-27632 or blebbistatin, the myotube cell cluster was still able migrate, but became strongly elongated with long interconnecting cell processes as expected for a tissue under stretch. As consequence, the cell cluster showed large gaps between individual cells, which strongly increased in the total size over time (see quantification in Fig. 7h). Consistently, RNAi-mediated depletion of both the regulatory light chain of the myosin II (*spaghetti squash*, *sqh*) and the myosin II heavy chain (*zipper, zip*) phenocopies the pharmacological inhibition of Rok (Fig. 7d, g, Supplementary Movie M21; quantification in Fig. 7h). Migratory defects and the inability to tighten up the cell cluster finally led to small adult testes with reduced coils and bulky tips with numerous large holes in the muscle sheet similar to those depleted of Rho1 (compare Fig. 7k, l with Supplementary Fig. 1b, k). In conclusion, these data show an important role of Rok-driven actomyosin contractility in collective myotube migration. Together, our data do not support that actomyosin-dependent contractility is required for myotube forward movement, but rather contribute to the integrity of the migrating cell cluster.

## Discussion

### Myotube migration—a model system for collective cell migration

In this study, we established a model system for studying collective cell migration in organ culture that allows high-resolution long-term live-imaging microscopy combined with genetic, pharmacological, and mechanical perturbation analysis. Our data imply that a contact-dependent migration mechanism acts as a driving force to polarize *Drosophila* myotubes and to promote their directional movement along the testes. A contact-stimulated migration has been already observed in cultured cells many years ago, but the molecular mechanisms underlying this phenomenon has been never analyzed in more detail^45^. Thomas and Yamada observed that both primary neural crest cells and two neural crest-derived cell lines barely moved when isolated in suspension, but could be stimulated up to 200-fold to migrate following contact with migrating cells^45^. This process might help to ensure the cohesion and coordination of collectively migrating myotubes to form dense muscular sheets in the walls of developing hollow organs. Those muscle fibers that race ahead will immediately cease migration when they lose contact with their neighbors. That is exactly what we observed in our experiments. After ablation, an isolated myotube awaits restimulation by the other cells of the migrating cluster. Consistently, reduced N-cadherin function promotes single-cell migration toward the free space at the expense of collective directionality. The contact-dependent behavior of myotubes also resembles CIL, a well-characterized phenomenon^16^. CIL regulates the in vivo collective cell migration of mesenchymal cells such as neural crest cells by inhibiting protrusions forming within the cluster at cell–cell edges and by driving actin polymerization at their free edge^46^.

**Fig. 8:**
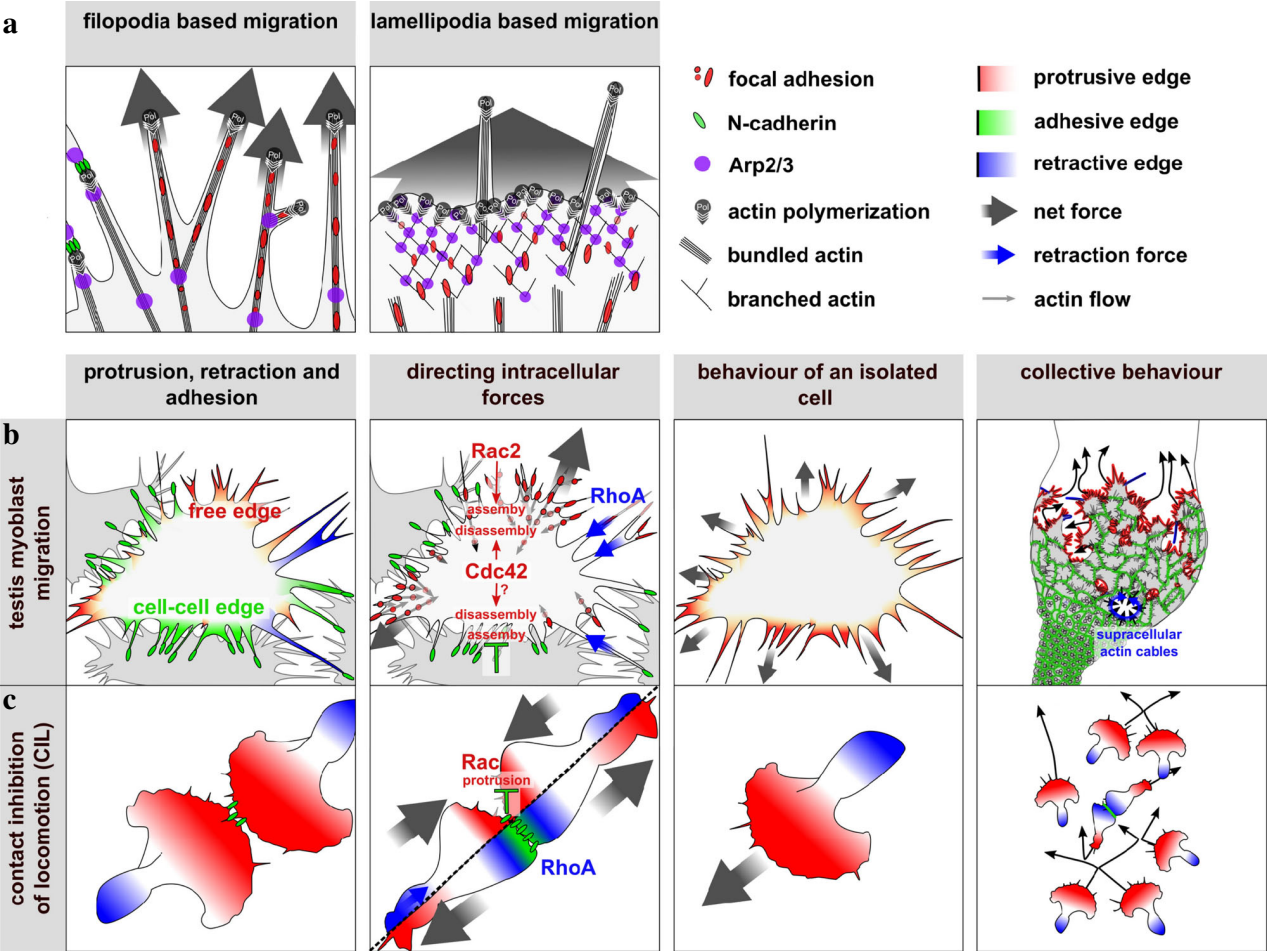
Proposed model. **a** Comparison between filopodia-based and lamellipodia-based cell migration. Lamellipodia-based migration requires the Arp2/3 complex generating branched actin filament networks that serve as the major engine to push the leading edge forward, whereas filopodia support mesenchymal migration by promoting cell–matrix adhesiveness at the leading edge stabilizing the advancing lamellipodium or by sensing the environment. In filopodia-based migration, it seems that filopodia replace the lamellipodium as the motor of motility. We assume that polymerization of bundled actin filaments through formins pushes parts of the membrane. Arp2/3 complex contributes to filopodia branching and thereby provides new barbed ends generating new filopodia. **b** Key features and cell behavior in testis myotube migration compared to **c** migrating mesenchymal neural crest cells undergoing CIL. Different from neural crest cells, myotubes did not migrate as loose cohorts, but maintain cohesiveness. Unlike neural crest cells, migrating myotubes are not simply polarized along a front-rear axis and do not form a contact-dependent intracellular Rho gradient that initiates cell polarization driving directed cell migration. In myotube migration, a contact-dependent asymmetry of cell–matrix adhesion rather acts as a major switch to drive locomotion toward the free space. Individually or loosely connected migrating cells, like neural crests cells are able to migrate persistently due to classical front-rear polarity. By contrast, testis myotubes rely on constant cohesion to break symmetry. Supracellular contractile actin cables contribute to the integrity of the migrating cell cluster and thereby to cohesion.

Different from neural crest cells, myotubes did not migrate as loose cohorts, but maintain cohesiveness (see model in Fig. 8). In the context of more-adhesive cells, a CIL-related mechanism, termed frustrated CIL has been proposed by which cell–cell junctions can determine the molecular polarity of a collectively migrating epithelial sheet^47,48^. The authors provided evidence that cell–cell junctions determine the molecular polarity through a network of downstream effectors that independently control Rac activity at the cell-free end and Rho-dependent myosin II light chain activation at cell–cell junctions^47,48^. At the first glance, myotubes do not show an obvious polarized cell morphology with prominent polarized protrusions. Instead, myotubes form numerous competing protrusions in all directions. However, protrusions pointing to the free space preferentially form more stable cell–matrix adhesions as anchorage sites for forward protrusions, whereas the lifetime of cell–matrix adhesions at cell–cell contacts is decreased. Thus, a contact-dependent asymmetry in matrix adhesion dynamics seems to be important for the directionality of migrating myotubes, a molecular polarity that has been also found in neural crest cells undergoing CIL^13,49^. Only when one of the adhesions of competing protrusions disassembles, pulling of the cell body toward the competing protrusions might contribute to symmetry breaking and directionality of collective migration (see model in Fig. 8).

### Rho GTPases differentially regulate myotube migration

We further provide evidence for a differential requirement of the Rho GTPases, Rac2, and Cdc42 in regulating cell–matrix adhesion. *cdc42* knockdown cells formed less cohesive clusters and showed a significant increase of cell–matrix adhesion lifetime probably due to a decrease cell–matrix adhesion turnover. In contrast, Rac2 depletion resulted in a prominent loss of cell–matrix adhesions, a phenotype that has already been described in Rac1^−/−^ mouse embryonic fibroblasts^50^. Thus, we propose a model in which cell–matrix adhesions are downregulated at N-cadherin-dependent cell–cell contacts, a process that requires Cdc42 functions. To finally test whether a contact-dependent reduction of cell–matrix adhesion in filopodia is sufficient to explain the observed collective cell behavior, we developed a simplified simulation model with a few rules governing cell behavior such as protrusive filopodia, matrix adhesion, cell–cell adhesion, and membrane resistance (Supplementary Movie M22). Unlike comparable computer models^51,52^, single cells do not possess directional information. A cell’s position is defined by the geometric center of all its filopodia, whose emergence/disappearance/elongation causes translation of the centroid, perceived as motion. Upon cell–cell contact, filopodia lose their cell–matrix adhesion and thereby their grip on the ECM, but keep connections through cell–cell adhesions. These adhesions are recognized by both contributing cells to calculate their respective centroids (see Supplementary Material). Using these simple rules, we could indeed model myotube collective migration, provided that cells are positioned in a confined area mimicking the unfolded testis surface (Supplementary Movie M22 2A, 2B). If filopodia disappear directly after contact, cells exhibit a different cell behavior that is very reminiscent of CIL (Supplementary Movie M22 2C). This simplified model further confirms our observation that local regulation of cell–matrix adhesion suffices to drive collective motility.

### Actomyosin function ensures the integrity of cohesive myotube cluster during migration

Myotube migration also requires Rho1 the *Drosophila* homolog of RhoA. Different from cells undergoing CIL, in migrating myotubes activated Rho1 was not enriched at cell–cell contacts between myotubes, but rather localized as local pulses along retracting filopodial protrusions at free edges. The effects of tensile forces have to be addressed separately in the future, by establishing one of the many existing force measurement techniques such as transition force microscopy or using in vivo FRET-based tensions sensors in this system. We show that loss of Rok activity, *sqh*, and *zip* phenocopies *rho*1 knock down suggesting that a canonical pathway controls myotube migration in which Rho1 acts through Rok kinase to activate myosin II contractility. This finding supports the notion that in testis myotubes, unlike many other cell types, locally restricted Rho-GTPase regulation outweighs global Rac/Rho regulation along the cell-rear axis to achieve directionality. Previous studies demonstrated that myosin II-dependent contraction is essential for coordinating the CIL response in colliding cells. In myotube migration, Rok-dependent actomyosin contraction seems to be not required to drive the myotube cluster forward, but rather contractile actin cables contribute to the integrity of the migrating cell cluster. Thus, myotube cluster behave more like a collectively migrating monolayered epithelial sheet during gap closure^53^. While myotubes migrate into any given free space, they leave larger gaps within the cell sheet surrounded by prominent circumferential actin cables. Constriction of these supracellular actin cables necessarily might lead to gap closure observed in wild type, but not in cells defective for RhoRok-driven actomyosin contractility.

### Filopodia-based myotube migration depends on the differential function of formins and the Arp2/3 complex

Efficient mesenchymal cell migration on two-dimensional surfaces is thought to^54^ require the Arp2/3 complex generating lamellipodial branched actin filament networks that serve a major engine to push the leading edge forward.

Interestingly, epithelial and mesenchymal cells form more filopodia when the Arp2/3 complex is absent^55–57^. Under these conditions, mesenchymal cells lack lamellipodia and adopt a different mode of migration only using matrix-anchored filopodial protrusions. Our data further provide evidence for a filopodia-based cell migration in a physiological context during morphogenesis. This migration mode largely depends on formin as central known actin nucleators generating filopodia^33,58^. Our data also suggest that the Arp2/3 and its activator, the WRC, contribute to a more efficient myotube migration by promoting filopodia branching, and thereby increasing the number of cell–matrix adhesions, thus increased anchorage sites. Overall, filopodia-based migration enables the cell to regulate discrete subunits of membrane protrusions as an answer to the environment. The sum of filopodial protrusions adds up to a net cell locomotion that occurs similarly during lamellipodial migration (please compare to Fig. 8). Filopodial matrix adhesion complexes not only provide anchorage sites, but also allow cells to directly restructure their microenvironment by membrane-bound matrix proteases. There is indeed increasing clinical evidence suggesting filopodia play a central role in tumor invasion^27,59^. Similar to invading cancer cells myotubes rather migrate through a 3D microenvironment composed of extracellular matrix restricted by pigment cells from the outside of the testis. Thus, it will be interesting to determine to what extent extracellular matrix restructuring by metalloproteinases is required for myotube migration.

Taken together, our data suggest that contact-stimulated filopodia-based collective migration of myotubes depends on a CIL-related phenomenon combining features and molecular mechanisms described in mesenchymal and epithelial sheet migration as well. We propose a model in which contact-dependent asymmetry of cell–matrix adhesion acts as a major switch to drive directional motion toward the free space, whereas contractile actin cables contribute to the integrity of the migrating cell cluster.

## Methods

### *Drosophila* genetics

Fly husbandry and crossing were carried out according to the standard methods^60^. Crossings and all UAS-Gal4-based experiments including RNAi were performed at 25 °C. The following fly lines were used: *mef2*-Gal4^61^, *beatVC*-Gal4 (BL-40654), *htl*-Gal4 (BL-40669), *lbe*-Gal4 (BL-47974), UAS-LifeAct-EGFP (BL-35544), UAS-LifeAct-RFP (BL-58715), UAS-GFP nls (BL-4775), UAS-mcd8-RFP (BL-32219), UAS-myr-mRFP (BL-7119), focal adhesion sensor UAS-FAT-GFP,^28^ and RhoA-activity sensor Ubi-Anillin.RBD-GFP (kindly provided by Thomas Lecuit)^39^. All UAS-RNAi lines we used are summarized in Supplementary Table. CyO/Sco;TM2/TM6B was used as a tool for multistep crossings, control crossings were conducted using w^1118^ (BL- 3605).

### Immunohistochemistry and fluorescence staining

Adult and pupal testis fixation was carried out using 4% F-PBS for 20 min. Samples were incubated in primary antibody over night. PBS was used for all washing steps. The following antibody was used: anti-cadherin-N (1:500, DSHB DN-Ex #8). The following secondary antibodies were used: Alexa Fluor 488 (Molecular Probes). Alexa Fluor Phalloidin 568 (Molecular Probes) staining on pupal testes was carried out during the secondary antibody incubation for 2 h (1:1000 in PBS). Adult testes were stained overnight (1:1000 in PBS). DAPI (Molecular Probes) was performed for 10 min.

### Microscopy/4D live-cell imaging of testicular nascent myotubes

Fixed pupal testes were embedded in Fluoromount-G (SouthernBiotech) and imaged on object slides. Adult testes were imaged in live-culture dishes in PBS, to maintain their natural shape. Light micrographs were taken with a Leica M165 FC stereo microscope equipped with a Leica DFC7000 T CCD camera. All fluorescent microscopic stills were taken with a Leica TCS SP8 with an HC PL APO CS2 20×/0.75 dry objective. 4D live-cell imaging was performed on developing testes of 33 h APF pupae. Prepupae were collected in prepupae stage and timed 33 h at 25 °C^30^. Life imaging of pupal testes was performed in M3 Medium (Shields and Sang) containing 0.2 mg/ml insulin (Invitrogen), 10% FCS (Thermo Fisher Scientific), and 1x penicillin/streptomycin (Gibco) at room temperature^62^. Images were taken on a Zeiss Observer.Z1 with a Yokogawa CSU-X1 spinning disc scanning unit and an Axiocam MRm CCD camera (6.45 µm  × 6.45 µm). Long-term imaging was performed using a LD LCI Plan-Apochromat 25×/0.8 Imm Korr DIC oil-immersion objective over 7 h, with a z-stack every 5 min. Close-ups were taken with a C Plan-Apochromat 63×/1.4 oil-immersion objective over 2 h, with a z-stack every 2 min. Laser ablation of single cells on the testis was performed with a Rapp TB 355 laser.

### Chemical inhibitors

Live-imaging experiments with chemical inhibitors were performed. All inhibitors were pre-solved in DMSO and stored at −20 °C. The following inhibitors were used: CK666 (100 µM, Sigma-Aldrich), formin inhibitor SMIFH2 (10 µM, Abcam), para-nitro-blebbistatin (10 µM, Cayman Chemical), and Rok inhibitor Y-27632 (10 µM, Cayman Chemical).

### Data processing and quantification with Fiji

Filopodia angles were obtained by manually tracking filopodia tips using the Multiple Points tool. The center of mass was calculated in Fiji. Testes for single-cell analysis (marked with *beatVC*-Gal4 » lifeact-EGFP) were always oriented with the testis tip, the presumptive destination, pointing left (see Fig. 1m’). The angle of the vector between filopodia tip and center of mass was calculated in R using the package matlib. Rose plots where generated using the package ggplot2. Membrane length for filopodia density (number per µm membrane) was quantified with the Free Hand Line tool and R. For single-cell analysis of oriented images, points left of a virtual horizontal line crossing the center of mass (front) were compared to points on the right-hand side (rear, see Fig. 1k). For processing and quantification of adhesion defects on still images a ROI with a defined size (120 × 220 px) in the middle of the migrating sheet was chosen. To obtain a black and white image for further analysis, a threshold was set (min: 299, max: 300). The cell number inside the ROI was counted. All further values were assessed using the Analyze>Analyze Particles-Tool. Area per cell was derived from the total area/cell number. Free cell edge was derived from the sum of all perimeters, as they constitute the length of black-to-white border, which is tantamount to the free cell edge. The gap number was derived from the number of coherent particles, when black and white picture are inverted (see also Supplementary Fig. 2b, c). The size of gaps during life imaging was measured with Analyze>Analyze Particles-Tool, too. Only gaps larger than 20 µm^2^ were analyzed. Corrected total cell fluorescence was measured on sum projections using Fiji^63^.

### Data processing and quantification of 4D life image stacks

Manual tracking of migrating myotubes was performed using the spots module in the Imaris 9.3 software. For drift correction, the reference frame module was used. The *x*-axis was positioned as axis from the genital disc to the testis hub. Excel was used for all processing and quantification. For more details see also Supplementary Dataset 1.

Distance on *x* is defined as the difference between the *x* values of the same track at *t* = 0 and *t* = 7 h on unprojected and unsmoothed 3D data. It was used as a measuring tool instead of speed, as fluctuations in manual tracking strongly affects velocity especially in slow cells.

Neighbor permanency is defined as:

1 $$\frac{{{\mathrm{Number}}\;{\mathrm{of}}\;{\mathrm{remaining}}\;{\mathrm{neighbors}}\;{\mathrm{at}}\;{\mathrm{t}} = 7\;{\mathrm{h}}}}{{{\mathrm{Number}}\;{\mathrm{of}}\;{\mathrm{neighbors}}\;{\mathrm{at}}\;{\mathrm{t}} = 0}}$$

Neighbors are defined as the six closest cells to a given cell at *t* = 0. A value of 1 means that all neighbors were kept.

#### Smooth data

As the manual tracking process is fluctuation-prone, we developed a process, taking this uncertainty into account. The smoothing process takes every spot as the center of a 10 µm circle and finds the track with the smallest angles, through these areas. The process is reiterated 30 times. Weak phenotypes could potentially lead to false negative results, but false positive phenotypes get much less likely (summary and formula in Fig. 3a, data after processing in Fig. 3b).

### Mercator projection

1. An approximation of the central axis is performed by splitting the dataset in ten subsets along the *x*-axis. In every subset, *yz* coordinates of the center point are approximated by triangulation using the leftmost, rightmost, and uppermost points. A central axis is derived from the point of gravity of the first five subsets and the last five subsets. Based on that, the *x*-axis is moved with a rotation matrix. This process gets reiterated three times (summarized in Fig. 3b–c).
2. An *yz*-vector $\overrightarrow {{\mathrm{r}}_{\mathrm{n}}}$ from every point’s respective *yz*-coordinate to the *yz*-coordinate of the central axis is generated. Its magnitude is the radius$\left| {\overrightarrow {{\mathrm{r}}_{\mathrm{n}}} } \right|$ of this point. The maximal radius of all points is $\left| {\overrightarrow {{\mathrm{r}}_{{\mathrm{max}}}} } \right|$. The formula of the central angle θ depends on the position of the *yz* coordinates of every respective point.
  For *y*_n_ < *y*_axis_ and *z*_n_ > *z*_axis_ the formula is
  2 $${\uptheta} = 2 * {\uppi} - {\mathrm{cos}}^{ - 1}\left( {\frac{{\overrightarrow {{\mathrm{r}}_{\mathrm{n}}} * \left( {\begin{array}{*{20}{c}} 1 \\ 0 \end{array}} \right)}}{{\left| {\overrightarrow {{\mathrm{r}}_{\mathrm{n}}} } \right| * \left|\left( {\begin{array}{*{20}{c}} 1 \\ 0 \end{array}} \right)\right|}}} \right)$$
  For *y*_n_ > *y*_axis_ and *z*_n_ > *z*_axis_ the formula is
  3 $${\uptheta} = - {\mathrm{cos}}^{ - 1}\left( {\frac{{\overrightarrow {{\mathrm{r}}_{\mathrm{n}}} * \left( {\begin{array}{*{20}{c}} 1 \\ 0 \end{array}} \right)}}{{\left| {\overrightarrow {{\mathrm{r}}_{\mathrm{n}}} } \right| * \left|\left( {\begin{array}{*{20}{c}} 1 \\ 0 \end{array}} \right)\right|}}} \right)$$
  For *z*_n_ < *z*_axis_ the formula is
  4 $${\uptheta} = {\mathrm{cos}}^{ - 1}\left( {\frac{{\overrightarrow {{\mathrm{r}}_{\mathrm{n}}} * \left( {\begin{array}{*{20}{c}} 1 \\ 0 \end{array}} \right)}}{{\left| {\overrightarrow {{\mathrm{r}}_{\mathrm{n}}} } \right| * \left|\left( {\begin{array}{*{20}{c}} 1 \\ 0 \end{array}} \right)\right|}}} \right)$$
  A new *y*-coordinate is generated using the formula
  5 $$\frac{{\pi /2 - {\uptheta}}}{{\pi /2}} * \left| {\overrightarrow {{\mathrm{r}}_{{\mathrm{max}}}} } \right| * \pi /2$$
  (summarized in Supplementary Fig. 2c–e).
3. To correct the *x*-axis with respect to $\left| {\overrightarrow {{\mathrm{r}}_{\mathrm{n}}} } \right|$, all datapoints are sorted by *x*-coordinate. ${\mathrm{x}}_{\mathrm{n}}^1$ is the *x*-coordinate of a given spot before correction. ${\mathrm{x}}_{{\mathrm{n}} - 1}^1$ is the point preceeding this point. Its corresponding point after correction is ${\mathrm{x}}_{\mathrm{n}}^2$ (summarized in Fig. 3e, f).

For the very first point the formula is:

6 $${\mathrm{x}}_1^1 = {\mathrm{x}}_1^2$$

For all further points ${\mathrm{x}}_{\mathrm{n}}^1$ the formula is

7 $${\mathrm{x}}_{\mathrm{n}}^2 = {\mathrm{x}}_{{\mathrm{n}} - 1}^2 + \left( {{\mathrm{x}}_{\mathrm{n}}^1 - {\mathrm{x}}_{{\mathrm{n}} - 1}^1} \right) * \frac{{|\overrightarrow {{\mathrm{r}}_{{\mathrm{max}}}} |}}{{\left| {\overrightarrow {{\mathrm{r}}_{\mathrm{n}}} } \right|}}$$

Track speed mean was measured in motility lab using smoothed tracking data, in order not to quantify manual tracking inaccuracies.

#### Biased angle to *x*-axis

The usual biased angle method measures the bias toward a predefined point. As myotubes do not migrate toward a point, but along a defined axis, we measured the angle-distribution to the *x*-axis to analyze myotube directionality. As angles get strongly affected by speed, this method can only compare cells with the same distance on *x* value (summarized in Supplementary Fig. 3f). Rose plots were generated in R using the ggplot2 package.

#### Meandering distance

To compare the directionality of samples with different speeds, their meandering distance $|\overrightarrow {{\mathrm{d}}_2} |$ was measured according to the following formula. The median for all tracks on the testis was calculated

8 $$\overrightarrow {{\mathrm{d}}_1} = \left( {\begin{array}{*{20}{c}} {{\mathrm{x}}_{\mathrm{n}}} & - & {{\mathrm{x}}_{\mathrm{n}}} \\ {{\mathrm{y}}_{\mathrm{n}}} & - & {{\mathrm{y}}_{{\mathrm{mean}}\;{\mathrm{per}}\;{\mathrm{track}}}} \end{array}} \right)|\overrightarrow {{\mathrm{d}}_2} | = |\overrightarrow {{\mathrm{d}}_1} | * \frac{{\left| {\overrightarrow {{\mathrm{r}}_{\mathrm{n}}} } \right|}}{{|\overrightarrow {{\mathrm{r}}_{{\mathrm{max}}}} |}}$$

Cell–matrix adhesion lifetime was measured with the spots module of the Imaris 3.0 software on 2D maximum projections.

Cell distance over time between cells isolated by ablation was quantified in R based on Imaris tracking data using the packages matlib, reshape2, tibble, and beeswarm.

### Statistics and reproducibility

Descriptive data presented in images was replicated five times (Figs. 1g–i, 3d–h”, 4a–g, 6f–h, 7a, b, k, and Supplementary Fig. 1b–n). Micrographs reflecting the quantification of filopodia represent 20 images of cells from 10 testes (Fig. 1k–o), from 8 testes (Fig. 3i–k), and all cells from 20 testes (Fig. 3a–c). Long-term testis imaging was performed five times per genotype/treatment (Figs. 1d, e, f; 2a–g’, i–l; 4 h, h’, i, i’; 6a, b; 7c, d). Experiments regarding focal adhesions longevity and localization were repeated three times (Figs. 3l, m, 4j, k; 6k, l, m). Ablation of a single myotube was repeated three times (Fig. 3o). Ablation experiments to isolate a single cell (Fig. 5a–d) or a pair of cells (Fig. 5h, h’) were repeated five times. Stills to quantify the effects of N-cad RNAi on myotubes were taken from eight testes for WT and N-cad RNAi in adults and pupae (Supplementary Fig. 2b, c, i, j). All statistical tests were performed using Prism 7 (GraphPad). Multiple comparisons were done using parametric (one-way ANOVA) or nonparametric (Kruskal–Wallis test) tests and for single comparisons Welsh’s *t* test or Mann–Whitney test was used. Depending on normal distribution, assessed with the Shapiro–Wilk test, either parametric or nonparametric tests were used.

### Image processing and graphic editing

For image processing and graphic editing, the following software tools were used: Zen Blue (Zeiss), LasX (Leica), Fiji (ImageJ 1.51), Imaris 9.3 (Bitplane), Inkscape 0.91. R Studio 1.2.5042 (RStudio, Inc.), and packages therein mentioned above. For displaying cell tracks, motility lab was used (Miller, unpublished).

#### Computer simulation of testis myoblast behavior

The software was programmed using Unity 2019.2.2f1 (Unity Technologies). A single cell in this model (see also Supplementary Movie M22) is not simulated as a single agent but consists of multiple simulated protrusion points (black dots). Their geometrical center (centroid) is calculated constantly and constitutes the cells position. Protrusion points radially move away from the centroid, mimicking filopodia elongation, but must counter membrane resistance that gets the higher the farther away the point moves from the centroid. On its way, every protrusion point creates its own cell–matrix adhesions (red dots). They mediate a filopodium (=protrusion points and all its adhesions) static friction which is needed to counter membrane resistance. If membrane resistance is higher than adhesion, the entire filopodium gets translated toward the centroid. Protrusion points and cell–matrix adhesions have a lifetime. New protrusion points are generated in a fixed distance from the centroid (gray circle) where the local density is lowest to recapitulate our finding that there is no asymmetry in myotube filopodia assembly. When a protrusion point touches the adhesion radius (gray circle) of another cell it loses its cell–matrix adhesions mimicking the measured shortened lifetime of real cell–matrix adhesions. The protrusion point is then turned into an adhesion point (green dot) which is recognized by both cells as one of their protrusion points to calculate their respective centroids. For more details see also Supplementary Dataset 2.

### Reporting summary

Further information on research design is available in the Nature Research Reporting Summary linked to this article.

## Supplementary information

Supplementary Information

Description of Additional Supplementary Files

Supplementary Movie 1

Supplementary Movie 2

Supplementary Movie 3

Supplementary Movie 4

Supplementary Movie 5

Supplementary Movie 6

Supplementary Movie 7

Supplementary Movie 8

Supplementary Movie 9

Supplementary Movie 10

Supplementary Movie 11

Supplementary Movie 12

Supplementary Movie 13

Supplementary Movie 14

Supplementary Movie 15

Supplementary Movie 16

Supplementary Movie 17

Supplementary Movie 18

Supplementary Movie 19

Supplementary Movie 20

Supplementary Movie 21

Supplementary Movie 22

Supplementary Data 1

Supplementary Data 2

Reporting Summary

## Data Availability

The data that support the findings of this study are available within the article, Supplementary Information, or from the corresponding author upon reasonable request. The source data underlying Figs. 1f, l, n, o, 2a, b, d, f, h, 3c, I, j k, n, 4h’, i’, l–q, 5e–g, j, I, 6c–e, j, I, n, and 7e–h, j, I and Supplementary Figs. 2d–h, k, 3a, b, d, f, 2a–e, 3a–f, 4a–d, f, 5a–e, 6b–h, and 7a–e are provided as a Source Data file. All data are available from the corresponding author upon reasonable request. Source Data are provided with this paper.

## Source data

Source Data

## Code availability

The source code and tutorial are available in Supplementary Dataset 2 and at the GitHub repository: https://github.com/SebastianLieb/CellMovement.
