# Supplementary material for: Filopodia-based contact stimulation of cell migration drives tissue morphogenesis
Source: Nat Commun. 2021 Feb 4;12:791. doi: 10.1038/s41467-020-20362-2 (PMC7862658; doi:10.1038/s41467-020-20362-2)
Supplement: Supplementary file 1 — Supplementary Information [file 41467_2020_20362_MOESM1_ESM.pdf]

Supplementary Information

Filopodia-based contact stimulation of cell migration drives tissue morphogenesis

Maik C. Bischoff, Sebastian Lieb, Renate Renkawitz-Pohl and Sven Bogdan

Supplementary Figure 1-3

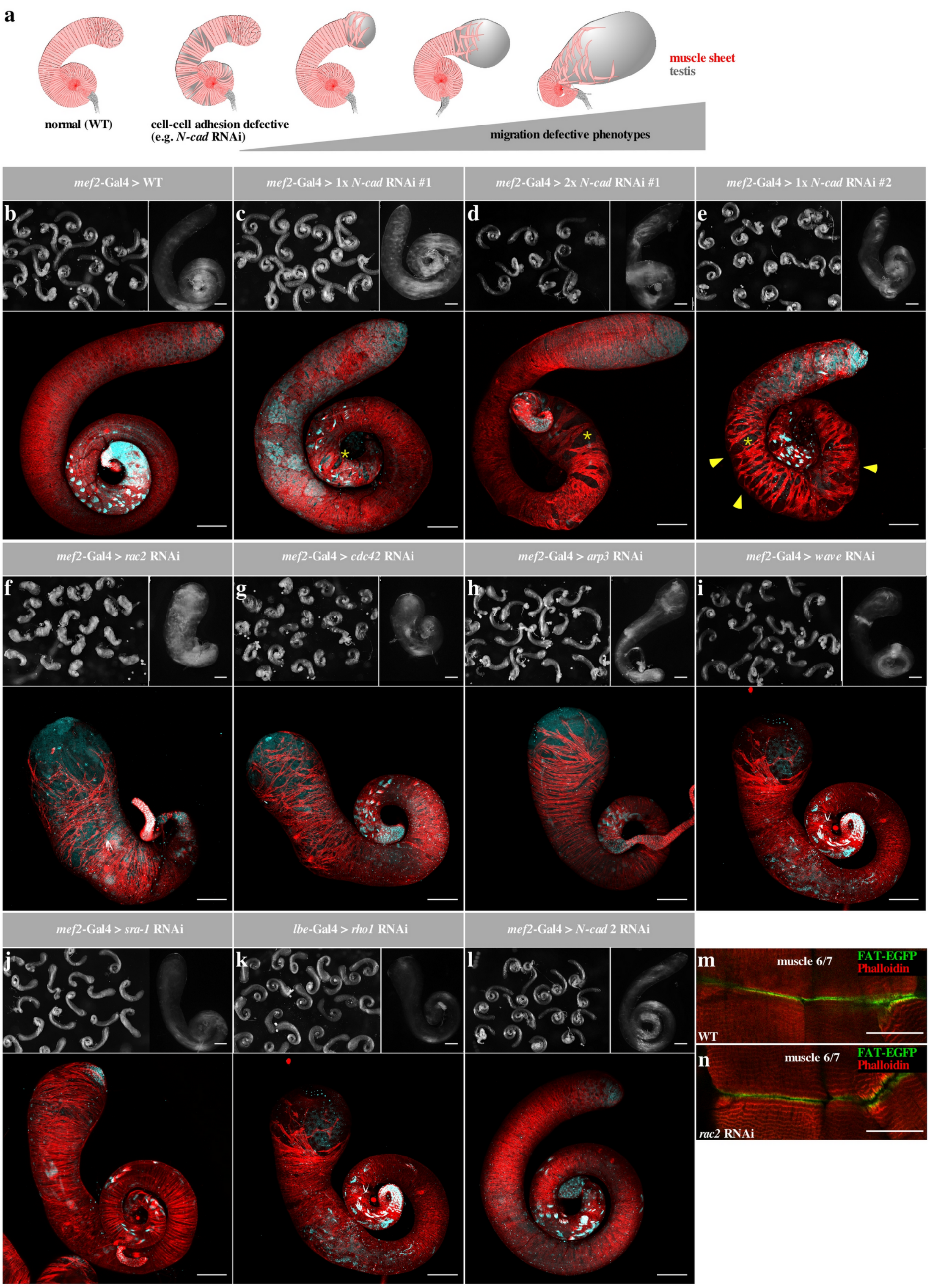

Supplementary Figure 1 Myotube migration resulted in an abnormal testis morphology

a Graphical representation of types of adult testis defects as a consequence of partial loss of adhesion or migration. **b-l** Adult testes with different genetic backgrounds. Upper left corner: light micrograph of several testis showing the phenotypic range, upper right corner: light micrograph of a single testis. Bottom: confocal image of a testis. muscle sheet stained with phalloidin (red) and nuclei stained with DAPI (cyan). **b** Wild type adult testis with a curled shape and an organized and entirely closed muscle sheet. **c, d** Expression of a *N-cad* RNAi transgene #1 driven by *mef2-Gal4* leads to small holes in the muscle sheet in a dose dependent-manner. **c** one copy of the RNAi transgene #1; d. two copies of the same RNAi transgene #1. **e** Expression of a stronger *N-cad* RNAi transgene #2 causes much stronger defects with large holes within the muscle sheet (yellow arrowheads). **f** *rac2* RNAi driven by *mef2-Gal4* leads to strong migration defects with a strongly dilated tip, partially uncovered, partially covered in disorganized muscles. **g** *cdc42* RNAi driven by *mef2-Gal4,* resembles *rac2* RNAi with slightly milder defects. **h, i, j** *arp3*, *wave* and *sra-1* RNAi driven by *mef2-Gal4,* leads to mild migration defects with a slightly dilated tip and small uncovered areas. **k** *rho1* RNAi driven by *lbe*-Gal4. Even using a weak driver line, prominent migration defects can be observed. **l.** *Ncad2* RNAi driven by *mef2-Gal4* resembles wild type testis without any defects. **m, n** Confocal images of FAT-GFP driven by *mef2*-Gal4 in larval body wall muscles 6/7. Muscle attachment sites, marked by FAT-GFP are not affected by *rac2* RNAi, indicating that *rac2* depletion has no general impact on matrix adhesion or integrin expression, but specifically affects cell-matrix adhesions in migratory cells. Using sibling flies, no cell-matrix adhesions can be detected in migrating myoblasts.

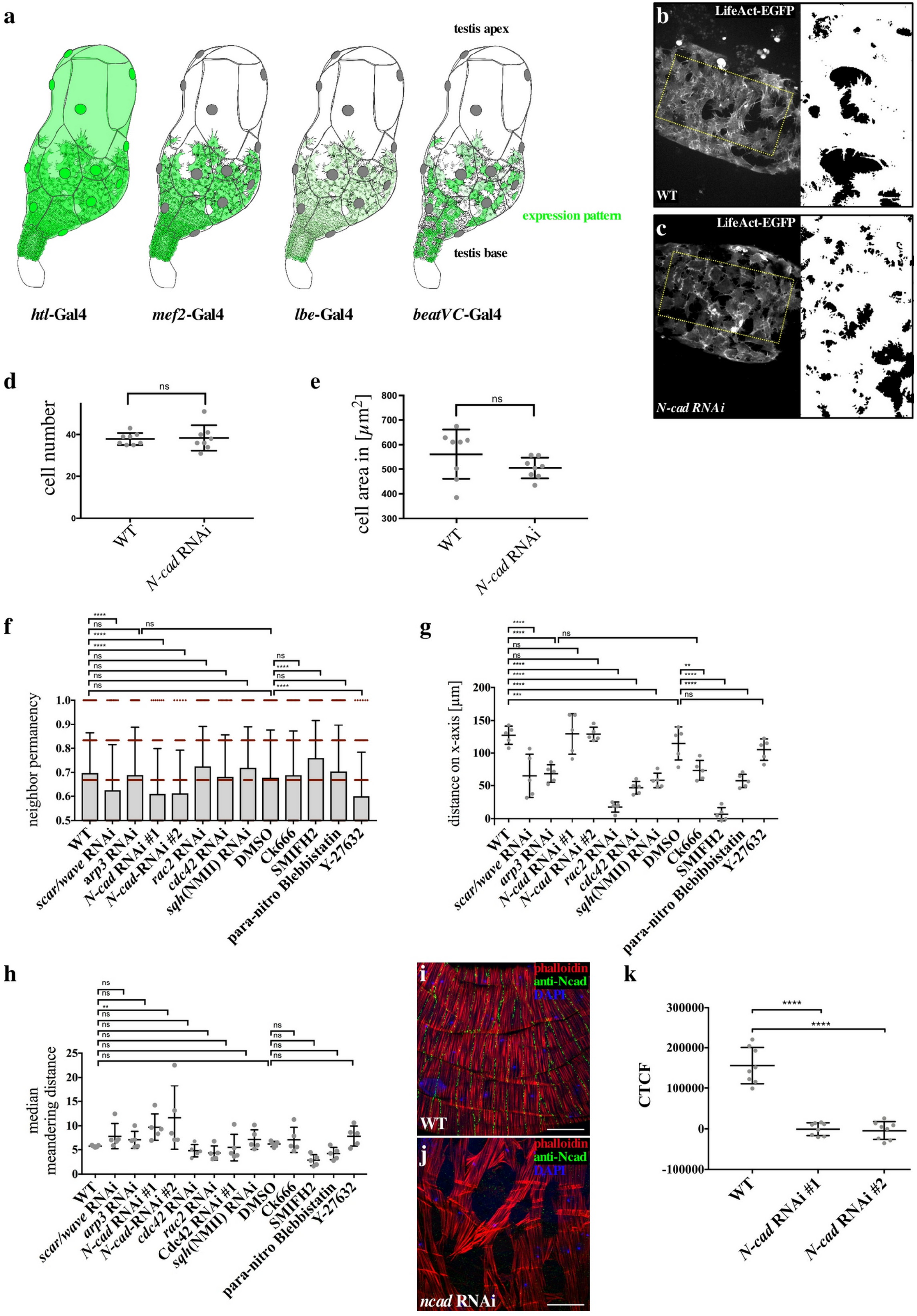

Figure S2 N-cadherin-dependent cell-cell contacts control the collective behavior of myotubes

a. Graphical representation of the expression patterns of the Gal4 driver lines used (in green). Compare to Fig. 1B. **b, c.** Overview and ROI's (120 x 220 px) in WT (B) and N-cad RNAi (C) on which quantification in D/E and Fig. 3E, F is based. Marked with yellow dashed lines in the overview. **d.** Cell number inside ROI's. Cell number is not affected upon *N-cad* RNAi. N=8 testes. Statistical testing: Unpaired t test (two tailed).d.-h, k. Data are presented as mean values ± SD. *P ≤ 0.05, ** P ≤ 0.01, *** P ≤ 0.001, **** P ≤ 0.0001. **e.** Area per cell inside ROI's. Cell Area is not affected upon *N-cad* RNAi. N=8 testes. Statistical testing: Mann-Whitney test (two tailed). **f, g, h, i.** Comparison of neighbor permanency (f.) N=5 testes with 130-506 cells. Statistical testing: Kruskal-Wallis test, distance on x-axis (g.), directionality based on biased angle (h.) and based on meandering distance (I), for all genotypes. g, h. N=5 testes. Statistical testing: One-way ANOVA. For every genotype, all trackable cells on 5 testes were analyzed. The number of tracks for every genotype equals the number of data points in h. **i, j.** Confocal images of adult testis muscle sheet stained with a specific anti-NCad antibody (green), phalloidin (red) and DAPI (blue). **i.** In wildtype (WT) Ncadherin localizes along the cell-cell junction. **j.** Expression of a *ncad* RNAi transgene strongly reduce anti-NCad immunostaining as quantified in **k.** for two independent RNAi transgenes. N=8 testes. Statistical testing: One-way ANOVA.

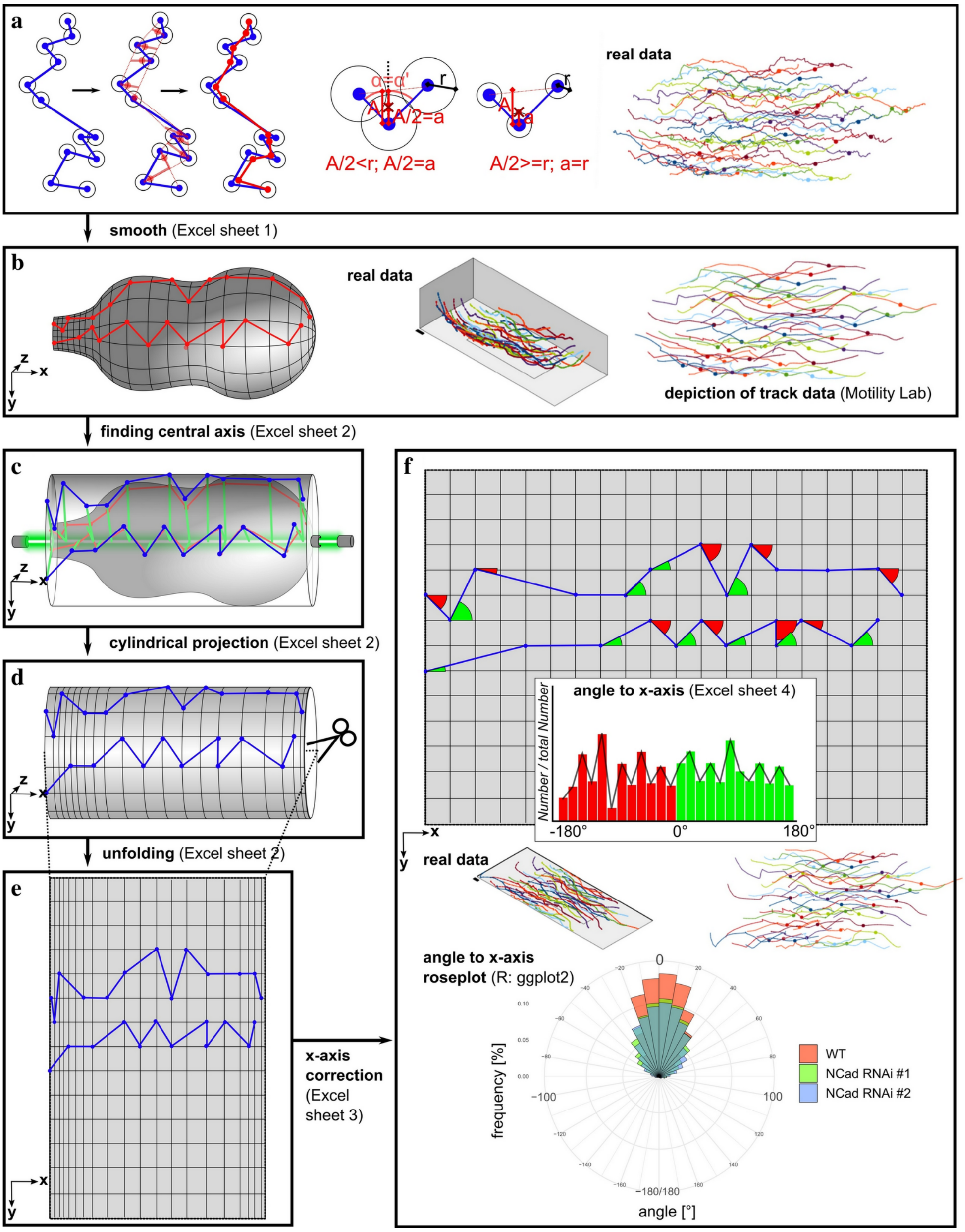

Supplementary Figure 3 Myotubes migrate on a two-dimensional surface

Myotubes migrate on the surface of an ellipsoid, thus on a two-dimensional surface, that is curved in space. This curvature did not allow to apply mathematical rules based in flat geometry. 3D migration tools do not consider the limitations of the surface, to which myotubes are bound but assume they can move freely. Instead, we developed a Mercator projection-based process, which allows for high angle-accuracy but neglects distances. **a -f** Steps of Mercator projection are shown (see material and methods for details).

Supplementary table Phenotypic strength using different Gal4 driver

A list of the RNAi transgenes used in this study, and phenotypic strength using different Gal4 driver lines.

effector	fly line ID	driver	phenotypic strength	Origin
**UAS-*Ncad RNAi***	v1092	*htl-Gal4*	defective adhesion	VDRC
	v1092	*mef-Gal4*	defective adhesion	VDRC
	v1093 (line #1)*	*htl-Gal4*	defective adhesion	VDRC
	v1093	*mef-Gal4*	defective adhesion	VDRC
	v101642 (line #2)**	*mef-Gal4*	defective adhesion	VDRC
**UAS-*Ncad 2 RNAi***	v101659	*mef-Gal4*	no phenotype	VDRC
	v36166	*mef-Gal4*	no phenotype	VDRC
**UAS-*arp3 RNAi***	v108951	*htl-Gal4*	medium migration defects	VDRC
	v108951	*mef-Gal4*	medium migration defects	VDRC
	v35258	*htl-Gal4*	medium migration defects	VDRC
	v35258	*mef-Gal4*	medium migration defects	VDRC
	BL-32921	*htl-Gal4*	no phenotype	BDSC
	v35260	*htl-Gal4*	no phenotype	VDRC
**UAS-arp2 RNAi**	v29944	*Mef-Gal4*	medium migration defects	VDRC
**UAS-*scar/wave RNAi***	NIG 4636R-1	*htl-Gal4*	medium migration defects	NIG
	NIG 4636R-1	*mef-Gal4*	medium migration defects	NIG
	BL-51803	*mef-Gal4*	medium migration defects	BDSC
	BL-36121	*mef-Gal4*	no phenotype	BDSC
	BL-31126	*mef-Gal4*	no phenotype	BDSC
**UAS-*sra1 RNAi***	BL-38294	*mef-Gal4*	medium migration defects	BDSC
**UAS-*rac2 RNAi***	NIG-8556R-1	*mef-Gal4*	strong migration defects	NIG
	NIG-8556R-3	*mef-Gal4*	strong migration defects	NIG
	v28926	*mef-Gal4*	no phenotype	VDRC
	v50349	*mef-Gal4*	no phenotype	VDRC
	v50350	*mef-Gal4*	no phenotype	VDRC
**UAS-*rac1 RNAi***	BL-28985	*mef-Gal4*	no phenotype	BDSC
	BL-34910	*mef-Gal4*	no phenotype	BDSC
	v49246	*mef-Gal4*	no phenotype	VDRC
**UAS-*mtl RNAi***	v108427	*mef-Gal4*	no phenotype	VDRC
**UAS-*cdc42 RNAi***	BL-28021	*htl-Gal4*	strong - medium migration defects	BDSC
	BL-28021	*mef-Gal4*	strong migration defects	BDSC
	v100794	*mef-Gal4*	medium migration defects	VDRC
**UAS-*rho1 RNAi***	v12734	*mef-Gal4*	lethal	VDRC
	BL-27727	*mef-Gal4*	lethal	BDSC
	BL-32383	*mef-Gal4*	lethal	BDSC
	BL-32383	*lbe-Gal4*	medium migration defects	BDSC
	v109420	*mef-Gal4*	no phenotype	VDRC
**UAS-*rhoL RNAi***	v102461	*mef-Gal4*	no phenotype	VDRC
**UAS-*dia RNAi***	v20518	*htl-Gal4*	strong migration defects	VDRC
	v20518	*mef-Gal4*	no phenotype	VDRC
	BL-25955	*mef-Gal4*	no phenotype	BDSC
**UAS-*capu RNAi***	v34278	*htl-Gal4*	no phenotype	VDRC
**UAS-*form RNAi***	v107473	*htl-Gal4*	no phenotype	VDRC
	v45594	*htl-Gal4*	no phenotype	VDRC
	v42302	*htl-Gal4*	no phenotype	VDRC
**UAS-*daam RNAi***	v24885	*htl-Gal4*	no phenotype	VDRC
**UAS-*frl RNAi***	v34412	*htl-Gal4*	no phenotype	VDRC
	v34413	*htl-Gal4*	no phenotype	VDRC
**UAS-*fhos RNAi***	v45837	*htl-Gal4*	no phenotype	VDRC
	v45838	*htl-Gal4*	no phenotype	VDRC
	v34034	*htl-Gal4*	no phenotype	VDRC
	v34035	*htl-Gal4*	no phenotype	VDRC
	v108347	*htl-Gal4*	no phenotype	VDRC
**UAS-*sqh* RNAi**	v7916	*mef-Gal4*	strong migration defects	VDRC
	v7917	*mef-Gal4*	medium migration defects	VDRC
	v109493	*mef-Gal4*	medium migration defects	VDRC
**UAS-*zip* RNAi**	v7819	*mef-Gal4*	strong migration defects	VDRC
**UAS-*mys RNAi***	v103704	*htl-Gal4*	lethal	VDRC
	v103704	*mef-Gal4*	lethal	VDRC
	v103704	*lbe-Gal4*	strong – no migration defects	VDRC

* shown in supplementary figure 1c; ** shown in supplementary figure 1d
