# Supplementary material for: Filopodia-based contact stimulation of cell migration drives tissue morphogenesis
Source: Nat Commun. 2021 Feb 4;12:791. doi: 10.1038/s41467-020-20362-2 (PMC7862658; doi:10.1038/s41467-020-20362-2)
Supplement: Supplementary file 2 — Description of Additional Supplementary Files [file 41467_2020_20362_MOESM2_ESM.pdf]

Description of Additional Supplementary Files

File Name: Supplementary Data 1
Description: Computer simulation and code.
