# Supplementary material for: Filopodia-based contact stimulation of cell migration drives tissue morphogenesis
Source: Nat Commun. 2021 Feb 4;12:791. doi: 10.1038/s41467-020-20362-2 (PMC7862658; doi:10.1038/s41467-020-20362-2)
Supplement: Supplementary file 25 — Supplementary Data 1 [file 41467_2020_20362_MOESM25_ESM.zip › computer simulation/Description.docx]

**Single cell behavior.** Simulated cells can be imagined as an elastic sheet, that stretches between points of protrusion (called *polymerization (pol.) point,* black dots) that each have their own direction vector. The cell body’s center is calculated as their geometrical center. *Pol. points* simulate the tips of real filopodia, where bundled F-actin gets elongated, pulling the membrane. In the computer model, *pol. points* create focal adhesions (FA’s, red dots) at regular intervals, simulating matrix tethering. They move away from the FA’s in a straight line that intersects the cell center (black line). FA’s have a lifetime, so that one *pol. point* can just have a limited number of them. Letting aside membrane force, FA’s are stationary. Membrane force is applied to all filopodia (=combination of *pol. points* and all its FA’s), pulling them inwards, toward the geometrical center. This force must counter the combined adhesive forces of FA’s in one filopodium. When the adhesive force is exceeded by membrane force, a filopodium will be translated towards the center of gravity. The membrane force grows with the length of all filopodia/cell (length=distance between point of gravity & *pol. point*). The relative amount of force applied to one filopodium equals its relative length compared to the other filopodia in the same cell, forcing longer filopodia to retract. When a certain threshold in relative length is met, *pol. Point.* get disassembled immediately, to simulate the effect of Rho1 (flashing blue). *Pol. Point.* have a lifetime. The algorithm spawns new *pol. Points.* in areas with a low density, working against filopodia asymmetry. As the membrane position is not simulated, new filopodia emerge on a radius around the gravity center, called *adhesion radius* (grey).

**Collective behavior.** As there is no collision model implemented, cells would simply migrate on tops (or through) each other, when colliding. We programmed a simple response to cell-cell contact. *Every pol. point* that touches the *adhesion circle* of another cell becomes an *adhesion point* and loses its FA’s, mimicking the drastically shortened lifetime of real focal adhesions at the cell-cell edge. The *adhesion point* is recognized by both cells to calculate their centroid. Its position will be at the closest point on the adhesion radius of the cell it adheres to.

Surprisingly, these cells, that are not simulated as an entity, but are the sum of all these small forces described above, behave like entities. Each filopodium can receive different information, so there is no model for establishing a front or rear “pole”. When spawned in a confinement, cells will start to evenly cover the limited area in a continuous sheet, resembling the behavior we observed with testis myoblasts.
