# Supplementary figures and images for: Filopodia-based contact stimulation of cell migration drives tissue morphogenesis
Source: Nat Commun. 2021 Feb 4;12:791. doi: 10.1038/s41467-020-20362-2 (PMC7862658; doi:10.1038/s41467-020-20362-2)

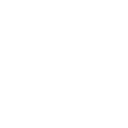

Supplement: Supplementary file 25 — Supplementary Data 1 [file 41467_2020_20362_MOESM25_ESM.zip › computer simulation/CellMovement 1.3 Source/Assets/Circle.png]

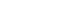

Supplement: Supplementary file 25 — Supplementary Data 1 [file 41467_2020_20362_MOESM25_ESM.zip › computer simulation/CellMovement 1.3 Source/Assets/DotLine.png]

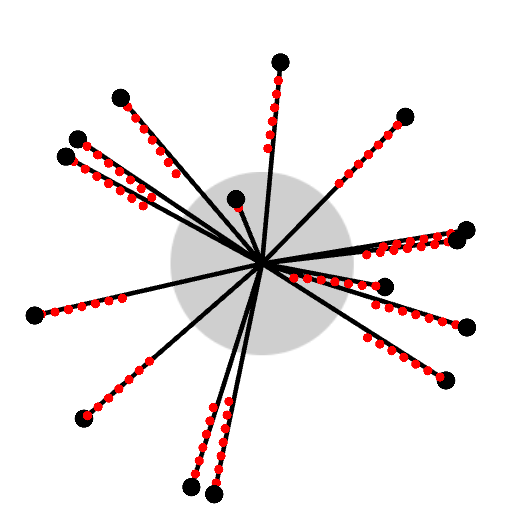

Supplement: Supplementary file 25 — Supplementary Data 1 [file 41467_2020_20362_MOESM25_ESM.zip › computer simulation/CellMovement 1.3 Source/Assets/Icon.png]

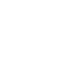

Supplement: Supplementary file 25 — Supplementary Data 1 [file 41467_2020_20362_MOESM25_ESM.zip › computer simulation/CellMovement 1.3 Source/Assets/PauseIcon.png]

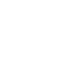

Supplement: Supplementary file 25 — Supplementary Data 1 [file 41467_2020_20362_MOESM25_ESM.zip › computer simulation/CellMovement 1.3 Source/Assets/PlayIcon.png]

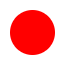

Supplement: Supplementary file 25 — Supplementary Data 1 [file 41467_2020_20362_MOESM25_ESM.zip › computer simulation/CellMovement 1.3 Source/Assets/RecordIcon.png]

## Pseudocode and Call Hierarchy

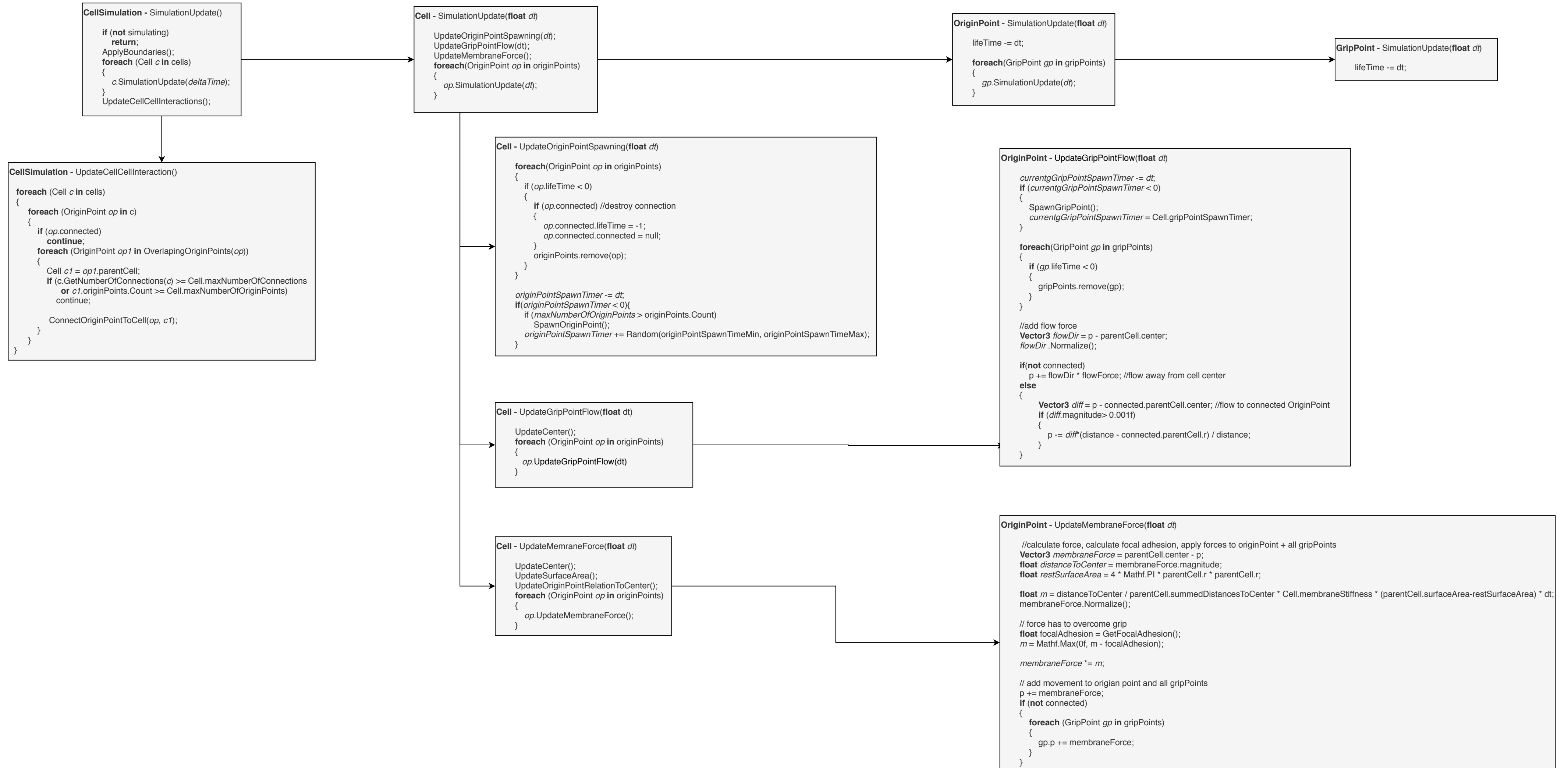

Supplement: Supplementary file 25 — Supplementary Data 1 [file 41467_2020_20362_MOESM25_ESM.zip › computer simulation/Pseudocode and Call Hierarchy.pdf]

# UML- Class Diagram

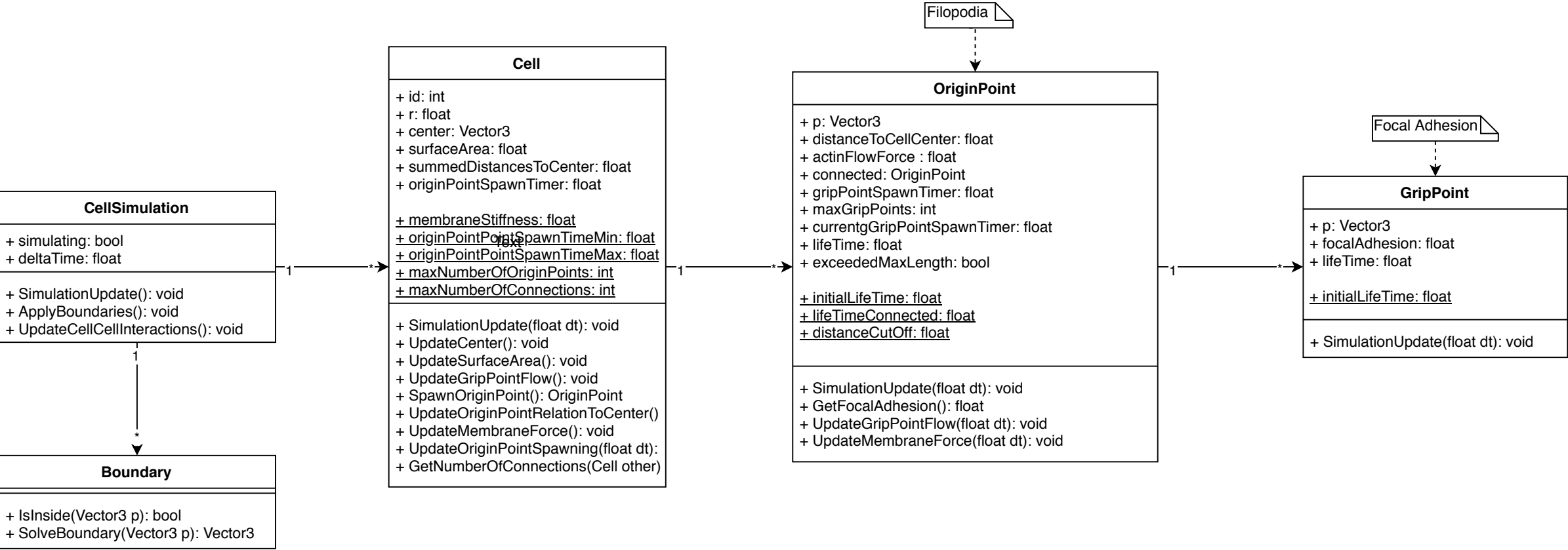

Supplement: Supplementary file 25 — Supplementary Data 1 [file 41467_2020_20362_MOESM25_ESM.zip › computer simulation/UMLClassDiagram.pdf]
