# Supplementary material for: Filopodia-based contact stimulation of cell migration drives tissue morphogenesis
Source: Nat Commun. 2021 Feb 4;12:791. doi: 10.1038/s41467-020-20362-2 (PMC7862658; doi:10.1038/s41467-020-20362-2)
Supplement: Supplementary file 27 — Reporting Summary [file 41467_2020_20362_MOESM27_ESM.pdf]

nature research

Corresponding author(s): Sven Bogdan

Last updated by author(s): 2020/11/04

Reporting Summary

Nature Research wishes to improve the reproducibility of the work that we publish. This form provides structure for consistency and transparency in reporting. For further information on Nature Research policies, see Authors & Referees and the Editorial Policy Checklist .

Statistics

For all statistical analyses, confirm that the following items are present in the figure legend, table legend, main text, or Methods section.

n/a	Confirmed	
☐	☒	The exact sample size (*n*) for each experimental group/condition, given as a discrete number and unit of measurement
☐	☒	A statement on whether measurements were taken from distinct samples or whether the same sample was measured repeatedly
☐	☒	The statistical test(s) used AND whether they are one- or two-sided<br>*Only common tests should be described solely by name; describe more complex techniques in the Methods section.*
☒	☐	A description of all covariates tested
☐	☒	A description of any assumptions or corrections, such as tests of normality and adjustment for multiple comparisons
☐	☒	A full description of the statistical parameters including central tendency (e.g. means) or other basic estimates (e.g. regression coefficient) AND variation (e.g. standard deviation) or associated estimates of uncertainty (e.g. confidence intervals)
☐	☒	For null hypothesis testing, the test statistic (e.g. *F*, *t*, *r*) with confidence intervals, effect sizes, degrees of freedom and *P* value noted<br>*Give P values as exact values whenever suitable.*
☒	☐	For Bayesian analysis, information on the choice of priors and Markov chain Monte Carlo settings
☒	☐	For hierarchical and complex designs, identification of the appropriate level for tests and full reporting of outcomes
☒	☐	Estimates of effect sizes (e.g. Cohen's *d*, Pearson's *r*), indicating how they were calculated

Our web collection on statistics for biologists contains articles on many of the points above.

Software and code

Policy information about availability of computer code

Data collection	Zen Blue 2 (Zeiss); Imaris 9.3 (Bitplane); LasX 3.5.2.18963 (Leica)
Data analysis	Fiji (Image J 1.51); Prism7 (GraphPad); Microsoft Excel 16.16.14, R Studio 1.2.5042 (RStudio, Inc.)

For manuscripts utilizing custom algorithms or software that are central to the research but not yet described in published literature, software must be made available to editors/reviewers. We strongly encourage code deposition in a community repository (e.g. GitHub). See the Nature Research guidelines for submitting code & software for further information.

Data

Policy information about availability of data

All manuscripts must include a data availability statement. This statement should provide the following information, where applicable:

- Accession codes, unique identifiers, or web links for publicly available datasets
- A list of figures that have associated raw data
- A description of any restrictions on data availability

Data availability

The data that support the findings of this study are available within the Article, Supplementary Information, or from the corresponding author upon reasonable request. The source data underlying Figs. 1f, l, n, o, 2a, b, d, f, h, 3c, I, j k, n, 4h’, i’, l, m, n, o, p, q, 5e, f, g, I, j, 6c, d, e, I, j, n, 7e, f, g, h, I, j and Supplementary Figs. S2d, e, f, g, h, k, S3a, b, f, –d, 2a–e, 3a–f, 4a–d, 4f, 5a–e, 6b–h, 7a–e are provided as a Source Data file. All data are available from the corresponding author upon reasonable request.

Field-specific reporting

Please select the one below that is the best fit for your research. If you are not sure, read the appropriate sections before making your selection.

- [x] Life sciences
- [ ] Behavioural & social sciences
- [ ] Ecological, evolutionary & environmental sciences

For a reference copy of the document with all sections, see nature.com/documents/nr-reporting-summary-flat.pdf

Life sciences study design

All studies must disclose on these points even when the disclosure is negative.

Sample size	Sample sizes were based on applicable standards and routinely employed sample sizes in the field. For genetic experiments involving crossing animals, each cross was set up at least three times. The sample sizes are indicated in the legends of each figure and were determined before the experiments were performed.
Data exclusions	No data were excluded.
Replication	All replicates are reported in the manuscript.
Randomization	Flies and pupa were chosen at random from the same genetic background. For all experiments we chose flies with the appropriate genotype. Among flies with the appropriate genotype flies were chosen at random. Animal controls were performed wherever possible. For each experiment, the experimental and control flies were collected, treated, and tested at the same time.
Blinding	Blinding was not feasible as pheneotypic strength and distinctive characteristics allows clear identification of the genotype in microscopic data. Blinding was not necessary as Excel was used for semi-automatic unbiased quantification of data based on experiments performed all by the same investigator.

Reporting for specific materials, systems and methods

We require information from authors about some types of materials, experimental systems and methods used in many studies. Here, indicate whether each material, system or method listed is relevant to your study. If you are not sure if a list item applies to your research, read the appropriate section before selecting a response.

Materials & experimental systems

n/a	Involved in the study	
☐	☒	Antibodies
☒	☐	Eukaryotic cell lines
☒	☐	Palaeontology
☐	☒	Animals and other organisms
☒	☐	Human research participants
☒	☐	Clinical data

Methods

n/a	Involved in the study	
☒	☐	ChIP-seq
☒	☐	Flow cytometry
☒	☐	MRI-based neuroimaging

Antibodies

Antibodies used	anti-NCadherin (DN-Ex #8; Developmental Hybridoma Bank; https://dshb.biology.uiowa.edu); AlexaFluor488 anti-rat (Molecular Probes)
Validation	anti-Cadherin-N; link to the website with all relevant information :https://dshb.biology.uiowa.edu/DN-Ex-8<br>Reference for usage in testis: Rothebusch Fender et al., (2017). Informations about Alexa Fluor Antibodies: https://www.abcam.com/

Animals and other organisms

Policy information about studies involving animals; ARRIVE guidelines recommended for reporting animal research

Laboratory animals	The following fly lines were used: mef2-Gal4 (Ranganayakulu et al., 1995), beatVC-Gal4 (BL-40654), htl-Gal4 (BL-40669), lbe-Gal4 (BL-47974), UAS-LifeAct-EGFP (BL-35544), UAS-LifeAct-RFP (BL-58715), UAS-GFP nls (BL-4775), UAS-mcd8-RFP (BL-32219), UAS-myr-mRFP (BL-7119), Focal Adhesion sensor UAS-fat-GFP (Nagel et al., 2017); RhoA-activity sensor Ubi-Anillin.RBD-GFP (Munjal et al., 2015). Age and sex are specified in detail in the methods. For the phenotypic analysis of testis defects only 33h APF (after puparium Formation) male pupa were used.
Wild animals	This study does not involve wild animals.

Field-collected samples	The study did not involve samples collected from the field.
Ethics oversight	Using Drosophila as a model system no ethical approval or guidance was required.

Note that full information on the approval of the study protocol must also be provided in the manuscript.
