# Supplementary material for: Filopodia-based contact stimulation of cell migration drives tissue morphogenesis
Source: Nat Commun. 2021 Feb 4;12:791. doi: 10.1038/s41467-020-20362-2 (PMC7862658; doi:10.1038/s41467-020-20362-2)
Supplement: Supplementary file 25 — Supplementary Data 1 [file 41467_2020_20362_MOESM25_ESM.zip › computer simulation/CellMovement(1).zip!/CellMovement/Description.docx]

We sought to create a computer simulation as a proof of concept for contact inhibition of singular protrusions being able to create motion without any sense of direction or position in the cell. In this software, cells are not simulated agents with a sense of direction but the sum of small-scale simulated processes. The simulated cells can be imagined as an elastic sheet, that stretches between focal points of protrusion. Hence, the cell body’s center is calculated as the point of gravity of all points of protrusion (called *polymerization (pol.) point*). *Pol. points* simulate the point in a real cell, where actin monomers assemble to F-Actin, elongating bundled actin-structures and pushing against focal adhesions, that are created on the way. In the computer model, *pol. points* create focal adhesions (FA’s) at regular intervals. They move away from the FA’s in a straight line, away from the center of gravity. After a certain time, FA’s will cease, so that one *pol. point* can just have a limited number of FA’s. Letting aside membrane force, FA’s are fixed in space, simulating their adhesion to the substrate.

These dynamics alone, would lead to an infinitely protruding cell, with longer and longer filopodia. Of course, an elastic membrane and a finite cell content will limit such an outgrowth. Membrane force is applied to all filopodia (=combination of *pol. points* and all its FA’s), pulling them inwards, toward the center of gravity. This force must counter the limited adhesive force of FA’s. Every FA adds an amount of adhesive force to a given filopodium. Not until its adhesive force is exceeded by membrane force, a filopodium will yield. Then, a translation of the entire filopodium with all its components towards the center of gravity occurs. The membrane force gets bigger with the length of all filopodia/cell (lengt=distance between point of gravity & *pol.point*). The relative amount of force applied to one filopodium equals its relative length compared to the other filopodia in the same cell. Due to that, longer filopodia get more membrane stress, forcing them to move inwards.

After some time such a cell would “equalize” between its pol. points, with all components canceling out each other. But after a certain time, filopodia (=combination of *pol. points* and all its FA’s) will disappear. New filopodia emerge at a random point, resulting in new asymmetry. As the membranes position is not simulated, new filopodia emerge on a radius around the gravity center, called *adhesion radius*. Cells with just the aforementioned behaviors will randomly “walk”, fueled by constant break of symmetry due to *pol. points* disappearing and emerging randomly.

Still, these cells have no program for contacting other cells. As there is no collision model implemented, they would simply migrate on tops (or through) each other, when colliding. We programmed a very simple response to cell-cell contact. *Every pol. point* that touches the *adhesion circle* of another cell will lose its FA’s, mimicking the drastically shortened lifetime of real focal adhesions at the cell-cell edge. At the same moment, this *pol. point* is also recognized by the other cell as its *pol. point*. After the switch, we call these mutual, non-FA-producing *pol. points,* *adhesion points*. *Adhesions points* are still taken into account to calculate the center of gravity. If membrane force gets higher than a certain value, the *adhesive point* will disappear.

Surprisingly, these cells, that are not simulated as an entity, but are the sum of all these small forces described above, behave like entities. If spawned on a limitless area, they will spread and not grow over each other, even tough, there is no collision model programmed. In that regard, their behavior resembles CIL, with the difference, that there is no polarization along an axis. Every filopodium can receive different information, so there is no model for establishing a front or rear “pole”. When spawned in a confinement, cells will start to evenly cover the limited area, resembling the behavior we observed with testis myoblasts. The dynamics observed here can be classified as a novel subtype of CIL with two main differences to the so far observed types of CIL. Firstly, inhibition does not occur on a “cell pole”-level. It is not a gradient shifting 180°. The inhibition occurs on a single filopodium-level. Eventually this leads to a very similar outcome, with the key difference, that these dynamics immediately cease to drive directionality when cells are separated, which does not allow for a conservation of direction over time. Secondly, cells keep being adhered to cells, they contacted once, ensuring even coverage of the area.
